# Selectivity of Explosives Analysis with Ambient Ionization Single Quadrupole Mass Spectrometry: Implications for Trace Detection

**DOI:** 10.1021/jasms.3c00305

**Published:** 2023-12-12

**Authors:** Simone Mathias, Marius Amerio-Cox, Toni Jackson, David Douce, Ashley Sage, Peter Luke, Richard Sleeman, Carol Crean, Patrick Sears

**Affiliations:** † School of Chemistry and Chemical Engineering, University of Surrey, Guildford GU2 7XH, U.K.; ‡ Waters Corporation, Stamford Avenue, Wilmslow SK9 4AX, U.K.; § Mass Spec Analytical, Future Space UWE North Gate, Bristol BS34 8RB, U.K.

## Abstract

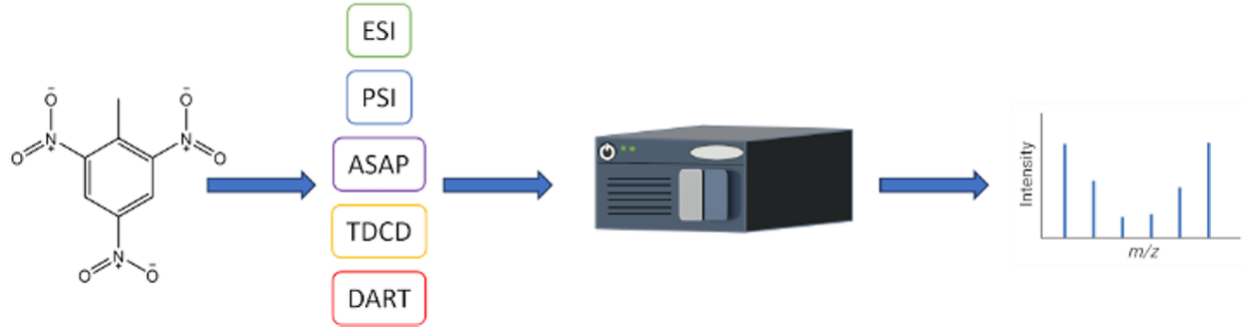

Ambient ionization (AI) is a rapidly growing field in mass spectrometry (MS). It allows for the direct analysis of samples without any sample preparation, making it a promising technique for the detection of explosives. Previous studies have shown that AI can be used to detect a variety of explosives, but the exact gas-phase reactions that occur during ionization are not fully understood. This is further complicated by differences in mass spectrometers and individual experimental set ups between researchers. This study investigated the gas-phase ion reactions of five different explosives using a variety of AI techniques coupled to a Waters QDa mass spectrometer to identify selective ions for explosive detection and identification based on the applied ambient ionization technique. The results showed that the choice of the ion source can have a significant impact on the number of ions observed. This can affect the sensitivity and selectivity of the data produced. The findings of this study provide new insights into the gas-phase ion reactions of explosives and could lead to the development of more sensitive and selective AI-based methods for their detection.

## 1 Introduction

A key area of research within the field of mass spectrometry (MS) is the development of novel ambient ionization (AI) techniques, which allow for the direct, rapid analysis of samples with minimal sample preparation steps required.^1^ The first AI techniques described in the early 2000s, direct analysis in real time (DART)^2^ and desorption electrospray ionization (DESI),^3^ have led to the development of a growing number of AI sources which exploit a variety of ionization mechanisms. Other examples include dielectric barrier discharge,^4,5^ corona discharge,^6,7^ laser-based^8,9^ and electrospray.^10,11^ There are multiple examples in literature in which AI techniques have been used in fields such as forensics,^12^ environmental,^13^ clinical,^14^ and pharmaceutical.^15,16^

Although these publications contain many examples of use of AI-MS, understanding the advantages and limitations for specific techniques is challenging due to the often “homemade” setups, differences in experimental design, and mass spectrometers used. Several validation studies have been carried out,^17−19^ including the British Mass Spectrometry Society (BMSS) interlaboratory study,^20^ demonstrating the potential for AI as a tool for routine analysis and addressing usual analytical concerns like robustness, reproducibility and matrix effects. The BMSS interlaboratory study concluded that ambient ionization techniques are able to ionize a range of compounds/molecules with good precision and repeatability when care is taken with the experimental set up and that the data produced can be improved significantly with an appropriate internal standard.

Studies have been conducted into the performance of MS systems, investigating the differences between manufacturers systems and different mass analysers.^21−23^ Given that most of the identified studies use different instruments, it is hard to decouple the impact of all the different components on the observed results. Differences observed between systems could arise from droplet drying in the heated region of the atmospheric pressure ionization, collisions in the semivacuum area, transfer of ions through the instrument, space-charging effects, or the resolution of the mass analyzer and could result in the production of alternative gas-phase ions or change the ratios of the ions produced. We therefore decided to standardize our experiments on a single instrument only changing the source conditions.

Explosive compounds are known to undergo characteristic gas-phase ion/molecule reactions which can lead to increased specificity in detection and has been demonstrated in a number of research articles, including a variety utilizing ambient ionization techniques.^7,24−26^ An extensive review article by Forbes and Sisco highlights the amount of research that has previously been carried out in the field for trace detection of explosives, however no comparison was made between the techniques.^27^ Much of the literature surrounding ambient ionization mass spectrometry uses DART for explosives analysis, likely due to it being one of the few commercially available techniques.^28−30^ There are, however, a number of publications using paper spray,^31,32^ DESI,^26,33^ thermal desorption,^34,35^ and low temperature plasma,^36,37^ to name a few, which have been used for explosives detection. We have previously demonstrated that differences in the species produced can be obtained by using additives within a spray solvent by changing the ionization process.^38^

Here we present a study exploring differences in gas-phase ion–molecule reactions for five explosives ionized by a range of different AI techniques all using a common mass spectrometer. The explosives chosen include the most common classes encountered (nitroaromatic, nitramine, nitrate ester, and peroxide). AI types investigated included electrospray ionization (ESI),^39,40^ DART,^2^ atmospheric solids analysis probe (ASAP),^6^ thermal desorption corona discharge (TDCD),^41,42^ and paper spray^43^ on a single quadrupole instrument. This approach is designed to ensure that differences observed in the ion chemistry can be directly attributed to the sample introduction and ionization mechanisms and not differences in the mass spectrometry or sample preparation. The aim of the study is to identify selective ions for explosive detection and identification based on the applied ambient ionization technique. The work presented in this study will be compared to literature examples produced by using these AI inlet types.

## 2 Experimental Section

### 2.1 Materials

**Scheme 1:**
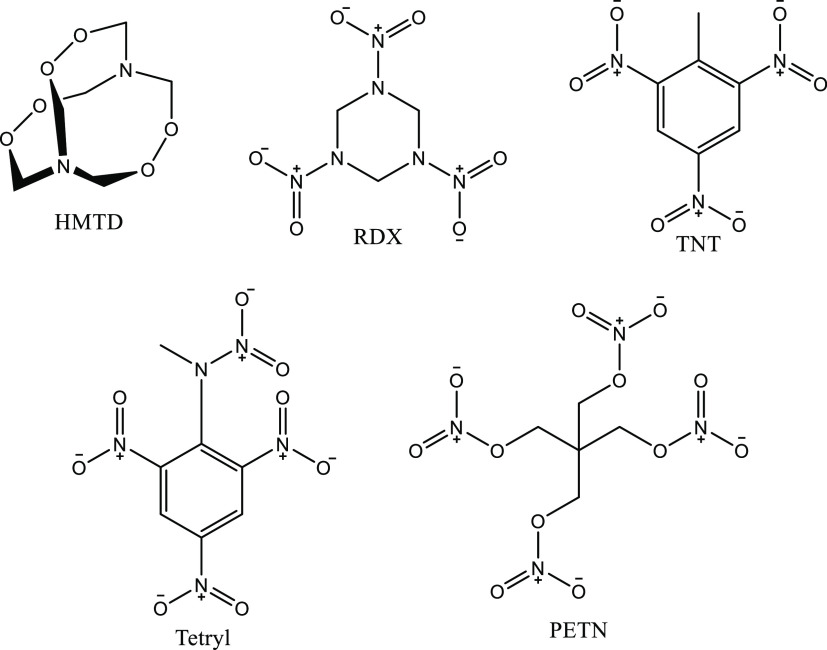
Chemical Structures of the Five Explosives Analyzed

Optima liquid chromatography mass spectrometry (LC-MS) grade methanol (MeOH), acetonitrile (ACN), isopropanol (IPA), formic acid, and water (H_2_O) were purchased from Fisher Scientific (Loughborough, UK). Pentaerythritol tetranitrate (PETN), 1,3,5-trinitro-1,3,5-triazinane (RDX), 2,4,6-trinitrotoluene (TNT), 2,4,6-trinitrophenylmethylnitramine (tetryl) and hexamethylene triperoxide diamine (HMTD) were purchased from AccuStandard (New Haven, CT, USA). Structures of the explosives are listed in Scheme 1. Ammonium chloride (NH_4_Cl), ammonium nitrate (NH_4_NO_3_), ammonium formate (NH_4_HCO_2_), ammonium acetate (NH_4_CH_3_CO_2_), and lithium acetate (LiCH_3_CO_2_) were purchased from Merck (Gillingham, UK). Helium gas (99.996% purity) was obtained from BOC (Guildford, UK).

Borosilicate glass melting point tubes were purchased from Fisher Scientific (Loughborough, UK) for use with ASAP. Itemizer sample traps (Teflon-coated fiberglass swabs) were purchased from DSA Detection (St. Albans, UK) for use with the thermal desorber. Whatman grade 1 chromatography paper 200 × 200 mm was purchased from Sigma-Aldrich (Gillingham, UK) for use with paper spray. Open Spot cards were obtained from IonSense (Saugus, MA, USA) for use with the DART.

### 2.2 Experimental Setup

A Waters Acquity QDa mass spectrometer (Waters, Wilmslow, UK) was used to obtain all of the data. The QDa instrument was operated in performance mode with an external rotary pump (Vacuubrand RE 6) and 0.2 mm aperture. MS was operated in full scan over the mass range *m*/*z* 30–500, with a scan time of 0.15 s. Positive mode was used for HMTD and negative mode was used for PETN, RDX, TNT, and tetryl. The cone voltage was varied from ±1–25 V and the source temperature was set to 150 °C. Details for individual ionization source experimental setups can be found below.

PETN, RDX, TNT, and tetryl were diluted in MeOH to a concentration of 100 μg/mL for use with AI techniques. The explosive HMTD arrived at the desired concentration of 100 μg/mL in ACN and was used as delivered.

All standards were diluted to 5 μg/mL for experiments carried out with the standard ESI source with 1 mM of either ammonium nitrate (PETN), ammonium chloride (RDX), ammonium acetate (TNT), ammonium formate (tetryl), or lithium acetate (HMTD) in 50:50 MeOH:H_2_O. The additives were selected based on the most abundant response for the most intense ion.

#### 2.2.1 Direct Injection by Electrospray Ionization

The standard ESI source was coupled to the QDa instrument to allow for direct injection experiments to take place. A Waters Acquity LC instrument without a column present was used to transfer samples to the ion source. A 50:50 MeOH:H_2_O solution was used to carry samples to the MS with a flow rate of 0.5 mL/min and an injection volume of 2 μL. Samples were prepared as shown in the Experimental Setup section.

#### 2.2.2 Paper Spray

An in-house constructed paper spray source (Ion Beam Centre, University of Surrey, UK) was connected to an external power supply (Stanford Research Systems, Sunnyvale CA, USA) and placed in front of the MS inlet. Sharp scissors were used to cut triangles of 1.6 cm × 2.1 cm (b x h) from the Whatman grade 1 chromatography paper. Aluminum foil was cut into rectangles 0.75 cm × 2 cm (*b* × *h*). Assembly of the paper spray substrate was carried out by folding the aluminum foil in half and placing the paper triangle within to ensure good contact between foil and paper as described by C. Costa et al.^44^ This was then attached to a glass slide and placed on a paper spray source. Photographs can be found in the Supporting Information (Figure S1) of the experimental setup used.

The analysis of PETN, RDX, TNT and tetryl was carried out using a spray solvent of 100% MeOH containing 0.1 mM NH_4_NO_3_ and NH_4_Cl. A spray solvent of 99.9% IPA and 0.1% formic acid was used for HMTD. A voltage of −2 kV was applied for negative ionization and +1.75 kV for positive ionization, respectively. Two μL of sample was placed onto the paper substrate and the solvent left to evaporate before 50 μL of spray solvent was added to the paper in addition the spray voltage applied.

#### 2.2.3 Atmospheric Solids Analysis Probe (ASAP)

A prototype ASAP source was obtained from Waters (Wilmslow, UK) and fitted directly to the QDa instrument. Nitrogen gas (2 L/min) was fed into the probe which was heated to 450 °C for negative ionization analytes and 200 °C for HMTD. The external power supply (Stanford Research Systems, Sunny Vale, CA, USA) was used to provide a discharge voltage (±3 kV) to the corona pin. Photo of the experimental set up can be found in the Supporting Information (Figure S2).

A Waters RADIAN mass spectrometer (Wilmslow, UK) was used to obtain ASAP spectra using air as the gas fed into the probe. All settings used above on the QDa instrument were applied to the RADIAN set up.

A borosilicate melting point capillary tube was dipped directly into the sample (mounted in a 3D printed sampled holder), and the solvent was left to evaporate before introduction to the ASAP source.

#### 2.2.4 Thermal Desorption Corona Discharge

A prototype thermal desorption corona discharge ionization source was obtained from Mass Spec Analytical (MSA, Bristol, UK) designed for use with QDa. The external power supply (Stanford Research Systems, Sunny Vale, CA, USA) was used to provide a discharge voltage (±3 kV) to the corona pin. The temperature of the heated elements of the source was controlled by a Rapid Analysis Controller (MSA, Bristol, UK) and was set to 275 °C for the analysis of PETN, RDX, TNT and tetryl and 150 °C for HMTD respectively. An external vacuum pump (Dwyer Instruments, Michigan City, IN, USA) set at ∼20 L/min by an external flow meter was used to help pull volatilized compounds from the swab surface into the ionization region provided by the corona discharge pin. A photo of the TDCD prototype is shown in Figure S3 in the Supporting Information.

One μL portion of sample was placed on a Teflon coated swab, and the solvent was left to evaporate before being placed into the TDCD ion source for analysis.

#### 2.2.5 Direct Analysis in Real Time

The DART source (Ion sense, Saugus MA, US) designed for QDa was used in conjunction with the Vapur interface (set at 1.3 turns). An OpenSpot module was fitted to the DART QDa source housing. Helium was used as the gas supply to create the metastable plasma for ionization, and nitrogen was used as the “standby” gas. The gas temperature was set to 150 °C for HMTD and 200 °C for the remaining explosives. A photo of the DART source can be found in Figure S4 in the Supporting Information.

One μL of sample was placed in the center of an OpenSpot card, and the solvent was left to evaporate before being introduced to the OpenSpot module and resulting heated helium plasma for ionization.

## 3 Results and Discussion

Five different explosive molecules were tested using ESI and ambient ionization methods; HMTD, PETN, RDX, TNT, and tetryl. The results are grouped by how each explosive behaved with respect to the different sample introduction methods.

### 3.1 HMTD

HMTD is an organic peroxide-based explosive which ionizes in positive mode. Table 1 reflects all ions detected for HMTD in order of abundance for the variety of ionization sources used within this study. The ions listed for paper spray were identified through a targeted approach using an extracted ion chromatogram which was compared to a blank due to the spectra containing high background noise. Spectra for ESI, ASAP (air), TDCD, and DART can be found in the Supporting Information in Figures S5–S8.

**Table 1:** Ions Observed during HMTD Analysis by Various Ionization Techniques in Order of Abundance

	ions observed in order of abundance (*m*/*z*)						
ionization technique	decreasing abundance →						
ESI^a^	213	229	215				
paper spray^b^	209	207	179	88			
ASAP (nitrogen)	209	179	145	88	207	191	
ASAP (air)	209	179	145	88	207	191	
TDCD	209	179	145	88			
DART	191	209	207	88	179	145	224

a With LiCH_3_CO_2_.

b IPA with 0.1% formic acid.

Previous research has shown that [M + H]^+^ ions are difficult to detect with ESI and better results are obtained by doping with alkali metal salts. In addition, HMTD readily undergoes oxidation in air to its dialdehyde form known as tetramethylene diperoxide diamine dialdehyde (TMDDD).^45^ This scenario was observed when using the ESI source with ions observed at *m*/*z* 213 and 229 (corresponding to [TMDDD + Li]^+^ and [TMDDD + Na]^+^ respectively). Sodium adducts are likely forming due to contamination from glassware, as it was not added to the spray solvent. It was not possible to generate any “in-source” fragmentation of these species. A very small amount of [HMTD + Li]^+^ was observed at *m*/*z* 215, which is unsurprising given that the ionization efficiency of TMDDD is greater than that of HMTD and the lack of separation used in this analysis.^45^

Although paper spray is nominally similar to ESI, when paper spray was used with a spray solvent containing IPA and 0.1% formic acid, it was possible to identify the [M + H]^+^ ion at *m*/*z* 209 along with the protonated TMDDD ion at *m*/*z* 207. A fragment of TMDDD was observed at *m*/*z* 179 relating to the loss of a carbonyl group and a characteristic fragment of HMTD was detected at *m*/*z* 88 corresponding to [C_3_H_6_NO_2_]^+^. Research conducted by M. Gonsalves et al. found that protonated HMTD was the most abundant ion when using paper spray when prepared in an acetonitrile/methanol/aqueous salt solution (1:1:2, v/v/v) containing 50 μM Li^+^, Na^+^ and K^+^.^32^ The researchers did not report any observation of fragments or presence of TMDDD when analyzing by paper spray.

**Figure 1:**
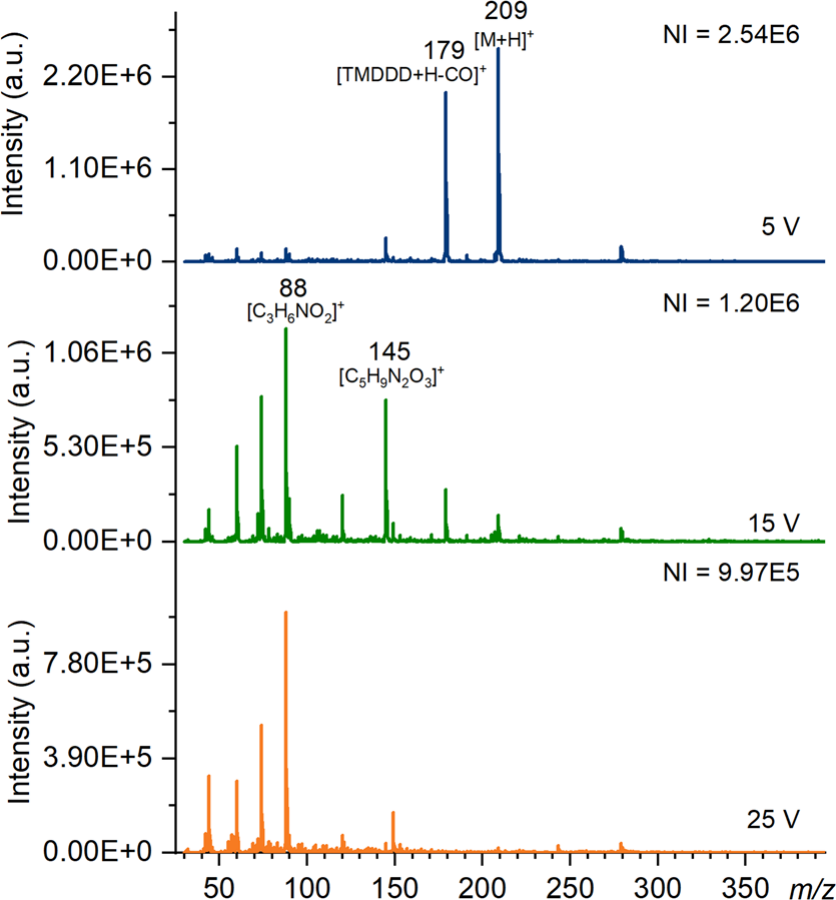
Mass spectra of HMTD produced using ASAP (nitrogen) on the Waters QDa at cone voltages of 5, 15, and 25 V in positive ion mode with the number of ions (NI) for the most abundant species. Major ions have been labeled, and known species identified.

**Scheme 2:**
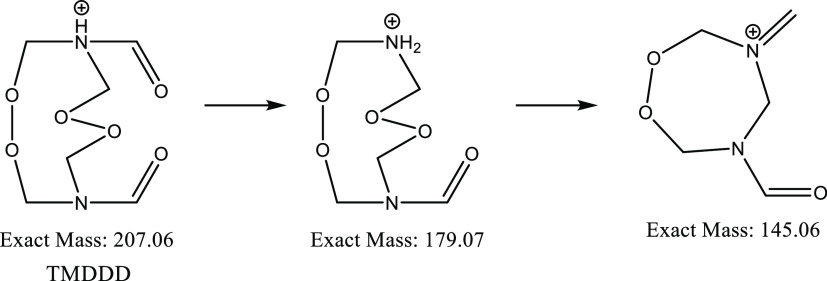
Structure of Protonated TMDDD and Potential Structures of Fragments with Exact Masses

Minor differences occurred in the ions produced by ASAP (using both nitrogen, seen in Figure 1, and air (Figure S6 in the Supporting Information) as the heated gas), TDCD and DART. The protonated HMTD ion was the most abundant ion detected using ASAP (air and nitrogen) and TDCD, followed by the TMDDD fragment at 179. Scheme 2 shows the structure of TMDDD and potential fragmentation structures. The third most significant ion was seen at *m*/*z* 145 and is characteristic of both HMTD and TMDDD, identified as [C_5_H_9_N_2_O_3_]^+^. In addition to the [TMDDD + H]^+^ ion at 207, the dehydration product ion of HMTD [HMTD – H_2_O] could be observed at *m*/*z* 191 by both ASAP and DART. Figure 1 demonstrates the effect of increasing cone voltage for analysis of HMTD by ASAP, as the voltage increases the intensity of the parent ion decreases while fragment ions tend to increase.

A unique ion at *m*/*z* 224 was identified using the DART source most likely to be [TMDDD + NH_4_]^+^ as ammonium adducts can form within the helium plasma from atmospheric conditions.^46^ A previous study conducted using DART attributed the ion at *m*/*z* 224 to [HMTD – H + NH_3_]^+^ and *m*/*z* 207 as [HMTD – H]^+^.^47^ Subsequent research using MS/MS of [TMDDD + NH_4_]^+^ identified a product ion at *m*/*z* 207 which further fragments to give the characteristic TMDDD ion at *m*/*z* 179.^45^ Further support for the identity of *m*/*z* 207 being [TMDDD + H]^+^ has been demonstrated by Oxley et al. and Dunn et al., who also used LC-MS/MS and accurate mass to provide evidence of this ions true identity.^48,49^ From the results obtained in this study, it is not clear whether the TMDDD is already present in the solution of HMTD or if it is forming within the ion source. Studies would need to be carried out using LC-MS to confirm where the formation is occurring, and this is a limitation of the ambient ionization techniques.

### 3.2 PETN

PETN is a nitrate ester explosive which ionizes in negative mode, and Table 2 lists all ions observed with various ionization sources. Spectra for ESI, paper spray, ASAP (nitrogen and air), and TDCD can be found in the Supporting Information in Figures S9–S13.

**Table 2:** Ions Observed during PETN Analysis by Various Ionization Techniques in Order of Abundance

	ions observed in order of abundance (*m*/*z*)					
ionization technique	decreasing abundance →					
ESI^a^	62	378	46			
paper spray^b^	378	351	353	62	315	46
ASAP (nitrogen)	378	62	362	315	46	
ASAP (air)	376	378	362	62	315	46
TDCD	378	376	362	315	62	46
DART	378	362	62	315	46	

a With NH_4_NO_3_.

b MeOH with NH_4_NO_3_ and NH_4_Cl.

PETN is commonly observed as its nitrate adduct at *m*/*z* 378 [M + NO_3_]^−^ or its parent ion at *m*/*z* 315 [M – H]^−^ by LC-MS.^50,51^ In all cases, except when using ASAP with air and ESI, the nitrate adduct at *m*/*z* 378 was the most abundantly detected ion relating to the explosive, which suggests that PETN is either more stable when in adduct form or has better ionizing properties compared to the parent ion. The nitrate adduct can form more readily by using additives such as ammonium nitrate, but can also form in the absence of additives through decomposition fragments of the nitrate ester itself.^50,52^

Chloride adducts [M + Cl]^−^ can also be observed when analyzing PETN by paper spray at *m*/*z* 351 and 353 due to the isotopes of ^35^Cl and ^37^Cl respectively. The order of abundance of detected ions observed in this study supports the research carried out by C. Costa et al. with the nitrate adduct being more relatively stable, with higher availability compared to the chloride adduct.^53^ Interestingly, the research published by Costa et al. and Tsai et al. did not report any observation of a parent ion at *m*/*z* 315 [M – H]^−^ suggesting that the type of mass spectrometer used (Thermo Orbitrap or LTQ vs Waters QDa) may impact the ions formed at the source.^31,53^ Previous research on a similar nitrate ester explosive (nitroglycerin) demonstrated that a dopant, such as dichloromethane can be useful for the production of chloride adducts [M + Cl]^−^ with DART.^2^

When using air as the heated gas in ASAP, an ion at *m*/*z* 376 identified as [M + CO_3_]^−^ (previously seen by Ostrinskaya et al.^54^ and Burns et al.^38^) was the most abundant and was also observed using the TDCD ion source which suggests that this adduct forms preferentially under atmospheric conditions.

**Figure 2:**
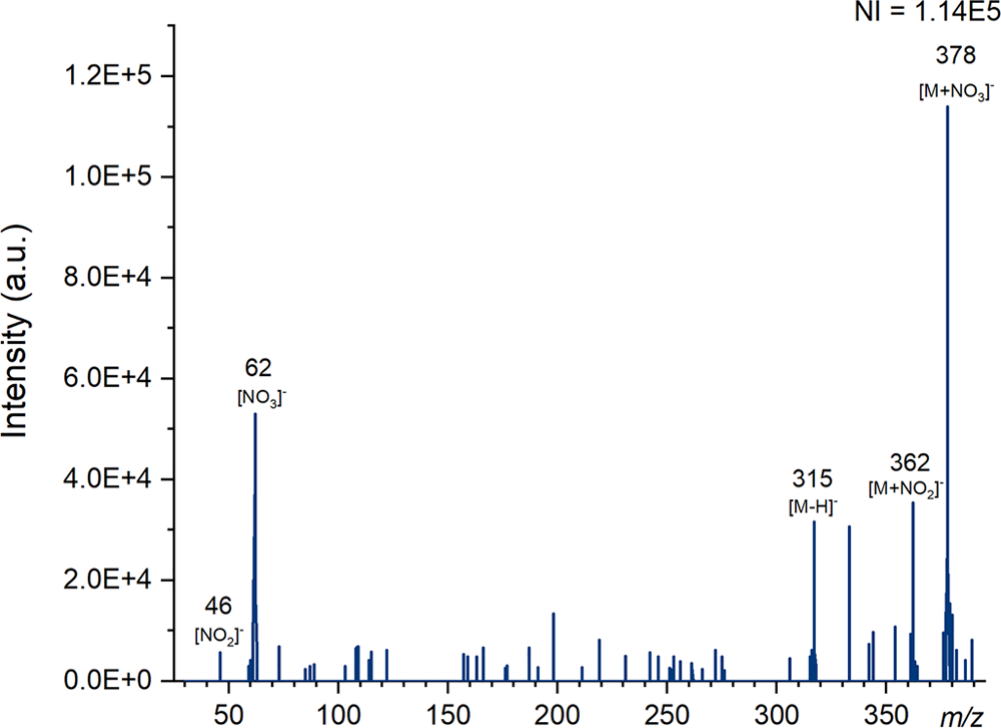
Mass spectra for PETN produced using DART on the Waters QDa instrument at a cone voltage of 5 V in negative ion mode with the number of ions (NI) for the most abundant species. Major ions have been labeled and known species identified.

A nitrite adduct ion [M + NO_2_]^−^ at *m*/*z* 362 was detected using ASAP, TDCD and DART (Figure 2), which has previously been reported by ASAP,^38^ low temperature plasma^36^ and LC-MS.^50^ This adduct potentially forms via degradation of intact PETN, or from NO_2_ in the atmosphere. Ions at 46 [NO_2_]^−^ and 62 [NO_3_]^−^ can be observed in a number of the spectra produced by these different ionization sources and we have previously shown that they occur in the background of negative ionization DART.^55^ The inclusion of a suitable additive could promote the formation of nitrate and nitrite adducts.

### 3.3 RDX

The nitroamine explosive known as RDX has been widely detected with a number of mass spectrometers and ionization techniques.^56−58^ Although a nitrogen containing molecule with a molar mass of 222.117 g mol^–1^, the deprotonated form [M – H]^−^ at *m*/*z* 221 is rarely reported in literature and is typically observed at low abundance compared to adduct ions.^59−61^Table 3 depicts the ions observed in this study with different ionization techniques for the analysis of RDX. Spectra for paper spray, ASAP (air), TDCD, and DART can be found in the Supporting Information in Figures S14–S17.

**Table 3:** Ions Observed during RDX Analysis by Various Ionization Techniques in Order of Abundance

	ions observed in order of abundance (*m*/*z*)							
ionization technique	decreasing abundance →							
ESI^a^	257	259	284	62	46			
paper spray^b^	284	62	257	259	46			
ASAP (nitrogen)	324	268	129	102	46	62	221	
ASAP (air)	268	283	324	129	102	62	221	46
TDCD	268	267	257	283	62	46		
DART	268	267	283	324	102	129	46	62

a With NH_4_Cl.

b MeOH with NH_4_NO_3_ and NH_4_Cl.

**Figure 3:**
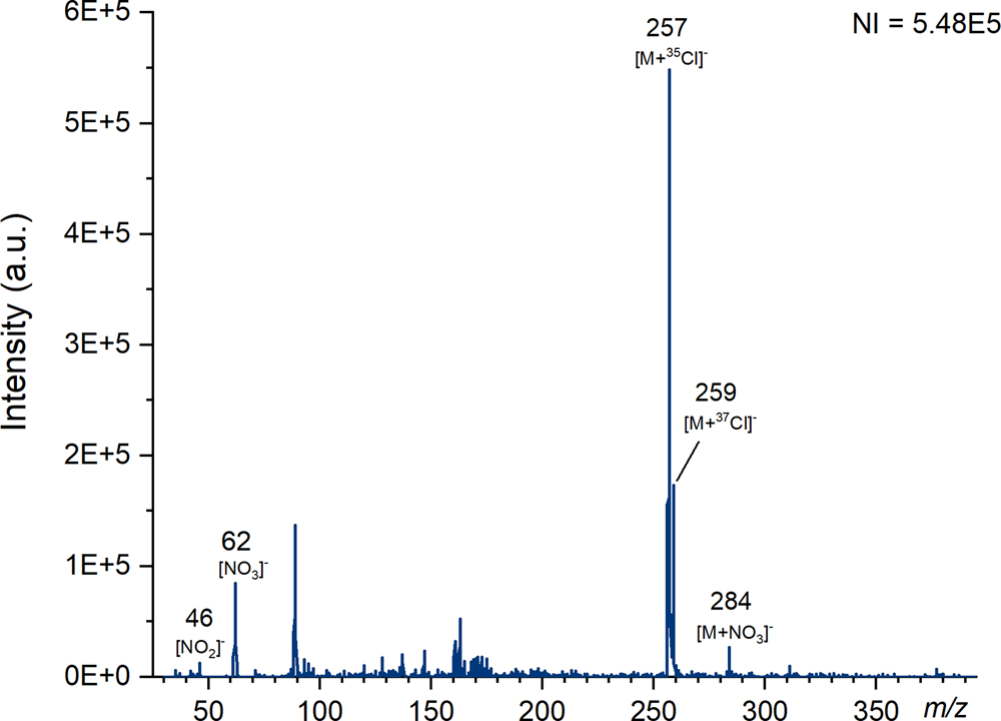
Mass spectra of RDX produced using ESI on the Waters QDa instrument at a cone voltage of 5 V in negative ion mode with the number of ions (NI) for the most abundant species. Major ions have been labeled and known species identified.

The results produced by ESI, shown in Figure 3, unsurprisingly show that the chloride adducts are the most abundant ions at *m*/*z* 257 and 259 corresponding to [M + ^35^Cl]^−^ and [M + ^37^Cl]^−^. For paper spray, it is noted that the ion at *m*/*z* 284, which can be attributed to [M+NO_3_]^−^ is more abundant than the chloride adducts, which further supports the finding of Costa et al.^53^ The spray solvent used for paper spray contained both ammonium nitrate and ammonium chloride, while for ESI only ammonium chloride was used. It has previously been reported that formation of nitrate adducts is possible when using ESI without the presence of additives for the analysis of RDX and may be generated due to impurities within the solvents.^62^

For ASAP (using air as the heated gas), TDCD, and DART, the nitrite ion [M + NO_2_]^−^ at *m*/*z* 268 is the most abundant and could be forming due to decomposition of RDX producing an NO_2_^–^ fragment or from the nitrite present in the atmosphere. An ion at *m*/*z* 267 is also detected by TDCD and DART, which has been assigned as [M + NO_2_ – H]^−^ by several studies.^59−61,63^ Since this assignment is widely accepted, it does not seem unreasonable to suggest that the ion at *m*/*z* 283 could be attributed to [M + NO_3_ – H]^−^ which has been identified in the spectra produced by ASAP (air), TDCD and DART, however only one article using atmospheric pressure photoionization mass spectrometry has made reference to this.^64^

**Figure 4:**
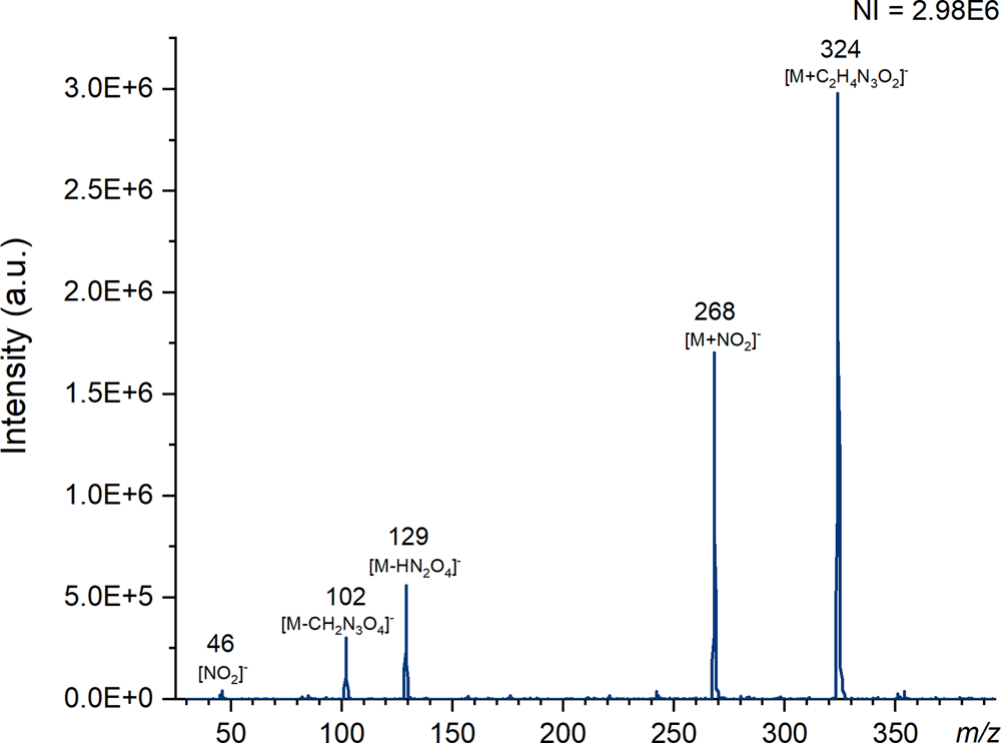
Mass spectra of RDX produced using ASAP (nitrogen) on the Waters QDa instrument at a cone voltage of 1 V in negative ion mode with the number of ions (NI) for the most abundant species. Major ions have been labeled and known species identified.

Known fragments of RDX at *m*/*z* 129 ([M – HN_2_O_4_]^−^) and 102 ([M – CH_2_N_3_O_4_]^−^)^65^ can be observed with ASAP, shown in Figure 4, and DART; however, the adduct ion at *m*/*z* 324 is not often reported in literature. It was proposed in 1996 that the ion may occur due to a reaction with a cyclic part of the structure.^66^ Liu et al. confirmed using DART-MS that recombination of the fragment ion at *m*/*z* 102 [C_2_H_4_N_3_O_2_]^−^ forms an adduct with intact RDX to give the ion at *m*/*z* 324 [M + C_2_H_4_N_3_O_2_]^−^.^67^ As shown in Figure 4, the increase in cone voltage leads to a decrease in the intensity of the adduct ions at *m*/*z* 324 and 268 and the fragment at *m*/*z*129.

### 3.4 Tetryl

The explosive tetryl, a nitroaromatic/nitromine, degrades to form *n*-methylpicramide and picric acid in aqueous conditions.^60,68,69^ The ion corresponding to *N*-methylpicramide (*m*/*z* 241*)* is a result of the loss of NO_2_ from tetryl [M – NO_2_]^−^ and is the most abundant ion observed using all nonspray ionization techniques as seen in Table 4. An ion at *m*/*z* 228 can be attributed to picric acid, and in the case of tetryl breaking down to this product it refers to [M – CH_3_N_2_O]^−^.

**Table 4:** Ions Observed during Tetryl Analysis by Various Ionization Techniques in Order of Abundance

	ions observed in order of abundance (*m*/*z*)										
ionization technique	decreasing abundance →										
ESI^a^	288	318	181	228	241	257	194	62	46	349	304
paper spray^b^	349	241	322	228	324	62	46				
ASAP (nitrogen)	241	228	213	181	194	286					
ASAP (air)	241	228	181	257							
TDCD	241	228	213	181	257	194	304	349			
DART	241	181	213	228	257	288	62	42	304	349	

a With NH_4_HCO_2_.

b MeOH with NH_4_NO_3_ and NH_4_Cl.

**Figure 5:**
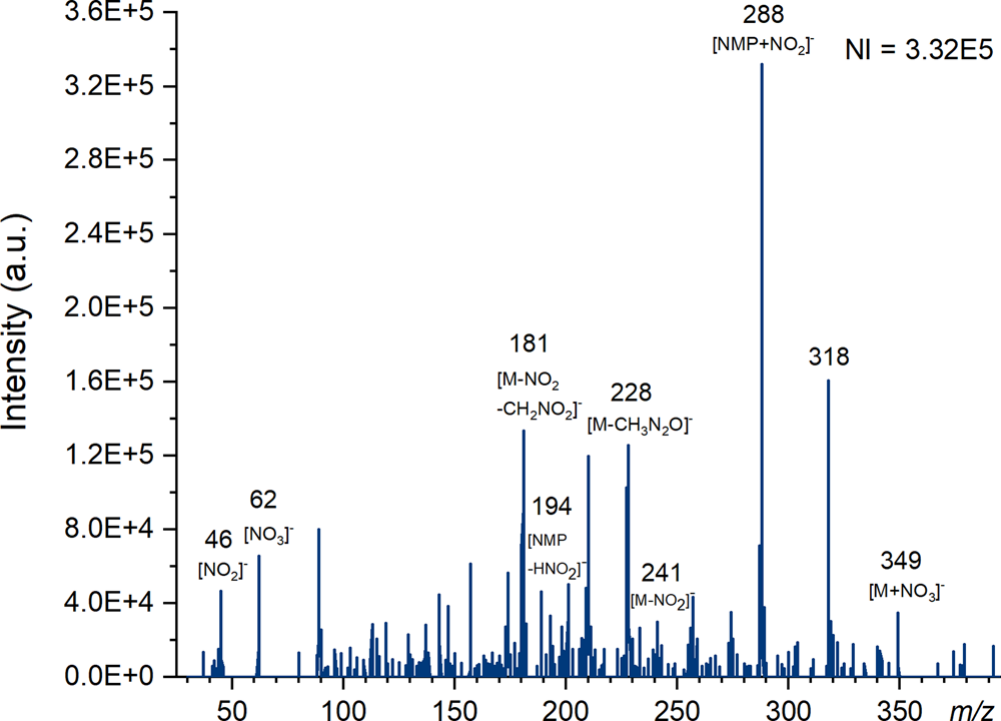
Mass spectra of tetryl produced using ESI on the Waters QDa instrument at a cone voltage of 5 V in negative ion mode with the number of ions (NI) for the most abundant species. Major ions have been labeled and known species identified.

When using ammonium formate as an additive with ESI (Figure 5), an ion at *m*/*z* 288 was the most abundant by far, and has been attributed to the nitrite adduct of *N*-methylpicramide (NMP) [NMP + NO_2_]^−^ rather than [M + H]^−^.^68^ Interestingly, this ion was also formed when DART was used as the ionization technique. The second most abundant ion observed in the tetryl ESI spectra was at *m*/*z* 318 and was assigned as [NMP + NO_2_ + NO]^−^ by Yinon et al.; however, Hubert et al. found that this ion was only observed in the presence of methanol and used high resolution mass spectrometry to identify the ion as an adduct of methanol and tetryl and assigned the formula [M + CH_3_OH – H]^−^.^60,68^ The exact identity of the species observed in these experiments is unclear due to the low resolution of the QDa mass spectrometer. Figure 5 shows that increased cone voltage causes a number of ions, particularly at *m*/*z* 288 and 318, to decrease in intensity but appears to have little effect on the picric acid (*m*/*z* = 228). Spectra for paper spray, ASAP (nitrogen and air), and DART can be found in the Supporting Information in Figures S18–S22.

**Figure 6:**
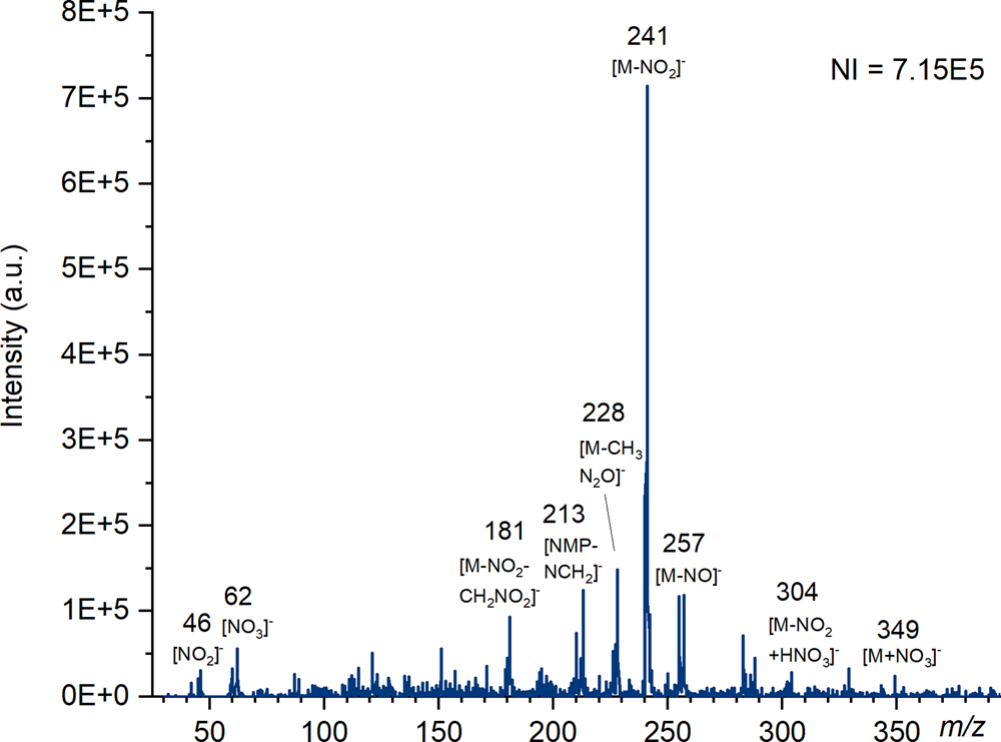
Mass spectra of tetryl produced using TDCD on the Waters QDa instrument at a cone voltage of 1 V in negative ion mode with the number of ions (NI) for the most abundant species. Major ions have been labeled and known species identified.

A fragment ion at *m*/*z* 181 was detected with all ionization techniques, except paper spray, and can be attributed to [M – NO_2_ – CH_2_NO_2_]^−^ and has been reported by users of low temperature plasma and Yinon et al.^36,60,70^ The loss of NO from tetryl results in the fragment ion at *m*/*z* 257 [M – NO]^−^ and is observed using ASAP with air, TDCD (Figure 6) and DART only. Interestingly, fragment ions at *m*/*z* 194 and 213, which have been previously reported as [NMP – HNO_2_]^−^ and [NMP – NCH_2_]^−^, respectively, both can be observed in spectra produced by ASAP (nitrogen) and TDCD, while the latter ion is present in spectra produced by DART.^60^Figure 6 shows that cone voltage does not significant affect the most abundant ion at *m*/*z* 241 below 25 V, however lower mass fragments increase in intensity as voltage increases.

Nitrate adducts are commonly seen at *m*/*z* 349 [M + NO_3_]^−^ using most of the AI techniques with the exception of ASAP. Nitrate and chloride were both included in the spray solvent for paper spray, and as expected, the nitrate adduct was more abundant than the chloride adduct. These results agree with those observed by Costa et al.^53^ A minor ion at *m*/*z* 304 often appears in spectra too and has been assigned [M – NO_2_ + HNO_3_]^−^.

### 3.5 TNT

Table 5 shows the ions observed when TNT, a nitroaromatic explosive, was analyzed by the ionization sources used in this study. Spectra for ESI, paper spray, ASAP (air), and TDCD can be found in the Supporting Information in Figures S21–S25.

**Table 5:** Ions Observed during TNT Analysis by Various Ionization Techniques in Order of Abundance

	ions observed in order of abundance (*m*/*z*)										
ionization technique	decreasing abundance →										
ESI^a^	226	62	227	46							
paper spray^b^	226	227	197	46							
ASAP (nitrogen)	227	210	197	151	167	124	139	137	120	181	105
ASAP (air)	227	243	226	213	197	210	167	151	167	124	
TDCD	226	197	227	213	243	210	167	137	62	46	
DART	226	197	260	227	213	243	242				

a With NH_4_HCO_2_.

b MeOH with NH_4_NO_3_ and NH_4_Cl.

The most abundant ion observed relating to TNT is the parent ion [M – H]^−^ at *m*/*z* 226, (particularly with spray-based ionization but also with DART and TDCD). The radical anion of TNT [M]^•-^ is favored by ASAP due to charge transfer occurring more favorably than proton abstraction.^38,60,71^ Since both ASAP and TDCD use a corona discharge for ionization, it would be expected that the same ions would be observed with both techniques, particularly for ASAP with air. It is possible that a difference is observed due to the following: (a) Concentration of the gas. In the ASAP source, the gas is focused to the end of the glass capillary rod, while in the TDCD the air is flowing through the heated region to pull the volatilized components toward the ionization region. (b) Impurities in the gas. As the TDCD used lab air, it may contain more moisture than the air used for ASAP (high pressure supply), which may explain the differences in ions produced. The amount of oxygen present in both air gas supplies should be equal and, therefore, should not affect the ionization. Additionally, the relative abundance of [M – H]^−^ and [M]^•-^ can vary depending on the experimental conditions using DART, as a previous study has shown the ion at *m*/*z* 227 [M]^•-^ as the base peak.^72^

**Figure 7:**
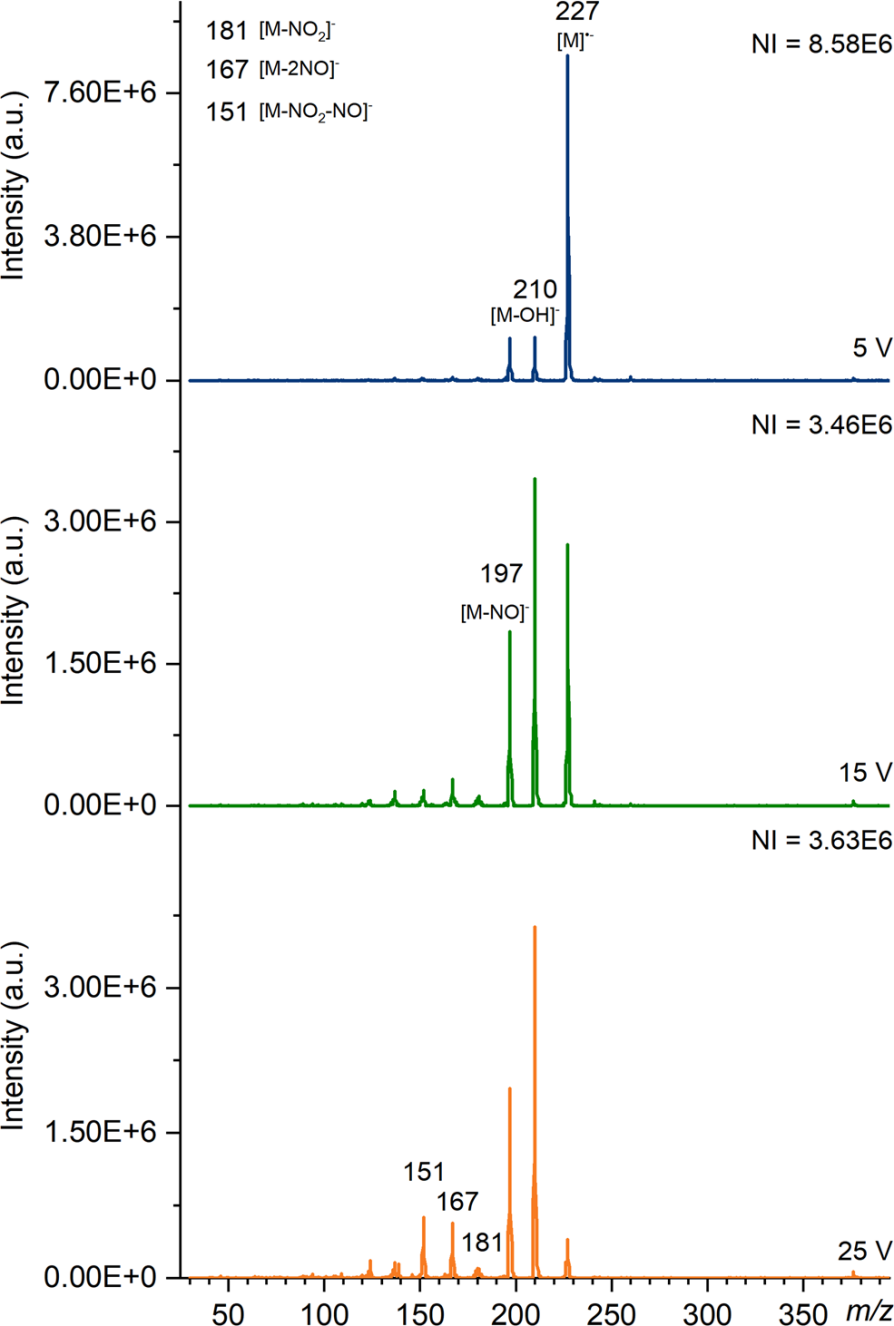
Mass spectra of TNT produced using ASAP (nitrogen) on the Waters QDa instrument at cone voltages of 5, 15, and 25 V in negative ion mode with the number of ions (NI) for the most abundant species. Major ions have been labeled and known species identified.

**Figure 8:**
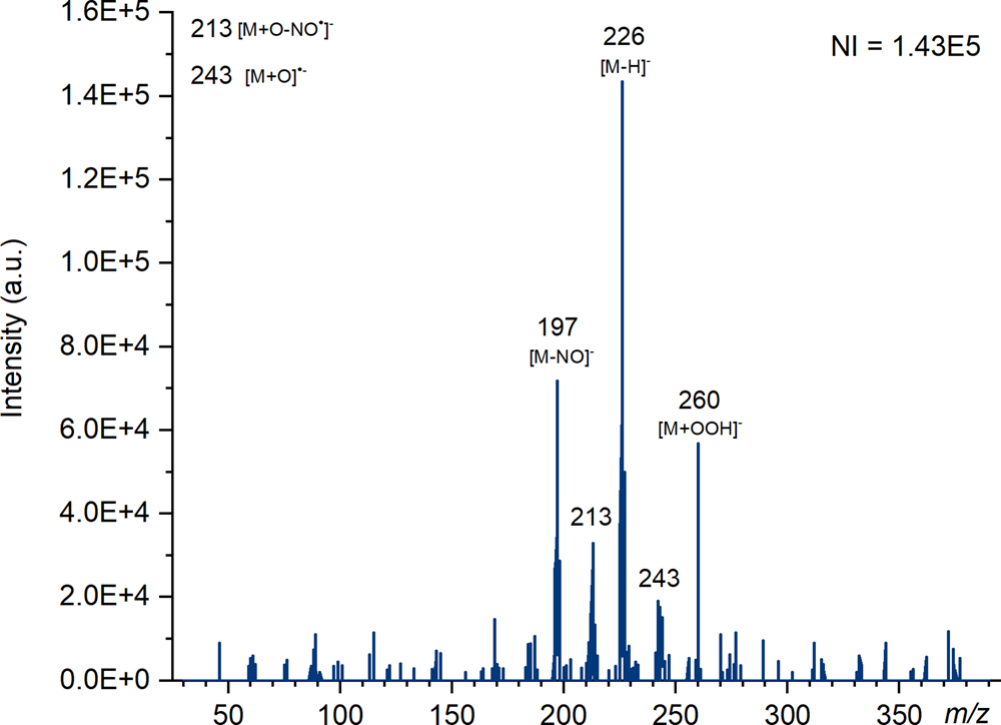
Mass spectra of TNT produced using DART on the Waters QDa instrument at a cone voltage of 1 V in negative ion mode with the number of ions (NI) for the most abundant species. Major ions have been labeled and known species identified.

Typical fragments of TNT were also detected with the majority of AI techniques at *m*/*z* = 210 [M – OH]^−^ and *m*/*z* = 197 [M – NO]^−^. ASAP with nitrogen, shown in Figure 7, produced a number of fragment ions as the cone voltage increased, including; [M – NO_2_ – NO]^−^ at *m*/*z* 151, [M – 2NO]^−^ at *m*/*z* 167, [M – 3NO]^−^ at *m*/*z* 137, [M – 2NO – HNO_2_]^−^ at *m*/*z* 120, [M – NO_2_]^−^ at *m*/*z* 181, and [M – 2NO_2_ – NO]^−^ at *m*/*z* 105.^73,74^ Two unidentified ions are seen at *m*/*z* 124 and 139 respectively, the latter was also observed by Boumsellek et al.^75^ A number of these ions were also observed using air as the desorption gas and also by TDCD, indicating a commonality between corona discharge type techniques. Paper spray and DART (Figure 8) were the exception to this, as a background ion was consistently present at *m*/*z* 210 using paper spray; therefore, it was not possible to determine the origin of this ion without using a MS with higher resolving power.

When air was used as the ionization gas for ASAP, some very interesting adducts and fragments were observed within the spectra, notably at *m*/*z* = 243 and 213. Due to the increased amount of oxygen present in the gas, an adduct [M + O]^•-^ forms, responsible for the ion at *m*/*z* 243.^73^ This oxidized form of TNT produces the fragment ion at *m*/*z* 213 [M + O – NO^•^]^−^.^76^ The oxidized form of TNT has also been detected by Song and Cooks using air plasma ionization, however they attribute the fragment at *m*/*z* 213 to [M – CH_2_O]^−^.^7^

Y. Song and R. Cooks also reported an ion at *m*/*z* 260 which they identified as a reaction product [M+OOH]^−^, which appears as a relatively major ion using DART. The minor peak at *m*/*z* 242 was reported as [M+O–H]^−^ by S. An et al. also observed with DART.^29^

### 3.6 Discussion

It appears that the chemistry of the explosive is the main factor responsible for the ions observed in most cases, with subtle changes occurring because of the ionization technique used. There are commonalities for the ions detected based on the ionization technique, notably when using ASAP, TDCD, and DART (all plasma-based), with the ionization mechanisms being related to APCI. Similarities also exist with ions produced by ESI and paper spray; here differences are likely a result of the experimental design, whereby different additives and solvents may have been used. There are also examples where the specific ion source changes the ion chemistry; TNT undergoing charge transfer or proton abstraction and the formation of the adduct ion at *m*/*z* 324 for RDX (only observed by ASAP and DART). Table 6 shows the most abundant ion observed for each of the ambient ionization techniques used with each explosive.

**Table 6:** Most Abundant Ion and Assignment Observed for Each Explosive with the Various Ambient Ionization Techniques Used

	paper spray	ASAP (N_2_)	ASAP (air)	TDCD	DART
HMTD	209 [M + H]^+^	209 [M + H]^+^	209 [M + H]^+^	209 [M + H]^+^	191 [M-H_2_O]^+^
PETN	378 [M + NO_3_]^−^	378 [M + NO_3_]^−^	376 [M + CO_3_]^−^	378 [M + NO_3_]^−^	378 [M + NO_3_]^−^
RDX	284 [M + NO_3_]^−^	324 [M + C_2_H_4_N_3_O_2_]^−^	268 [M + NO_2_]^−^	268 [M + NO_2_]^−^	268 [M + NO_2_]^−^
tetryl	349 [M + NO_3_]^−^	241 [M – NO_2_]^−^	241 [M – NO_2_]^−^	241 [M – NO_2_]^−^	241 [M – NO_2_]^−^
TNT	226 [M – H]^−^	227 [M]^•-^	227 [M]^•-^	226 [M – H]^−^	226 [M – H]^−^

While the sensitivity of the techniques has not been explored in a quantitative manner in this work, it can be noted that of the ambient ionization techniques ASAP and paper spray tend to produce the most intense signal for the explosive ions. While the sensitivity of the techniques has not been explored in a quantitative manner in this work, it can be noted that of the ambient ionization techniques ASAP and paper spray tend to produce the most intense signal for the explosive ions. There were no significant differences observed in the sensitivity of ASAP regardless of the gas used. The minimal blank signals (not shown) from ASAP suggest that it would have the highest signal-to-noise ratio of the techniques tested here (the blank signals of TDCD, DART, and paper spray were more significant). The overall sensitivity of the technique will depend on the chosen criteria for detection (most abundant ion, qualifier ions, etc.) which will also include aspects of the specific detector.

## 4 Conclusion

The work presented in this study suggests that the ion source used for explosive detection can change the selectivity (unique ions relating to analyte) and the sensitivity (number of ions observed) of the technique. This may be useful for researchers and/or analysts to consider when designing a method for explosive detection or for other molecules with similar chemistry. For example, when using ASAP with nitrogen a large number of fragment ions are typically observed; however, the use of air can introduce oxidative species (which could be used to increase specificity and hence improve confirmation of the compound). In addition, better selectivity may be achieved by using spray techniques, as adduct formation can be controlled through the inclusion of additives.

The data produced in this study using ESI via direct injection were carried out as a means of comparing the ions produced by ambient ionization techniques to a standard ionization method. Most laboratories would typically use a column as separation of mixtures is often necessary for improved detection rates, which can lead to increased analysis time and sample preparation. Ultrashort columns can be used to reduce analysis time;^77^ however, the cost of materials for LC-MS is significantly more expensive compared to ambient ionization sample introduction.

The main advantages of using ambient ionization revolve around rapid analysis time and minimal sample preparation; as a result ASAP, TDCD, and DART fit this criteria best due to their fixed geometry designs and commercially available sample introduction media. Paper spray is the exception to this, as the source used in this study was constructed in-house and required lining up with the MS inlet before each sample was analyzed and can result in failed sprays. A further limitation of paper spray is the process of preparing the paper triangles in the aluminum foil and glass slide; depositing the analyte and subsequently the spray solvent is more time-consuming compared to the other ambient ionization techniques used. Additionally, the spectra produced by ASAP tend to benefit from less interference from competing signals.

A higher resolution mass spectrometer would be required to explore some of the ions that could have multiple assignments to identify species with increased confidence.

## Data Availability

The data underlying this study are openly available in Open Science Framework at https://osf.io/nk5h8/. DOI: 10.17605/OSF.IO/NK5H8.

## Supporting Information Available

Figures showing experimental setup of ambient ionization techniques paper spray, ASAP, TDCD, and DART; mass spectra of explosives HMTD, PETN, RDX, tetryl, and TNT produced with ESI, paper spray, ASAP, TDCD, and DART (PDF)
