# Supplementary material for: Selectivity of Explosives Analysis with Ambient Ionization Single Quadrupole Mass Spectrometry: Implications for Trace Detection
Source: J Am Soc Mass Spectrom. 2023 Dec 12;35(1):50–61. doi: 10.1021/jasms.3c00305 (PMC10767746; doi:10.1021/jasms.3c00305)
Supplement: Supplementary file 1 — js3c00305_si_001.pdf [file js3c00305_si_001.pdf]

# Supporting information for

## The selectivity of explosives analysis with ambient ionization single quadrupole mass spectrometry: Implications for trace detection

Simone Mathias[1], Marius Amerio-Cox[1], Toni Jackson[1], David Douce[2], Ashley Sage[2], Peter Luke[3], Richard Sleeman[3], Carol Crean[1] and Patrick Sears[1*]

*Corresponding Author Email: p.sears@surrey.ac.uk

[1] School of Chemistry and Chemical Engineering, University of Surrey, Guildford GU2 7XH, UK [2] Waters Corporation, Stamford Avenue, Wilmslow SK9 4AX, UK [3] Mass Spec Analytical, Future Space UWE North Gate, Bristol BS34 8RB

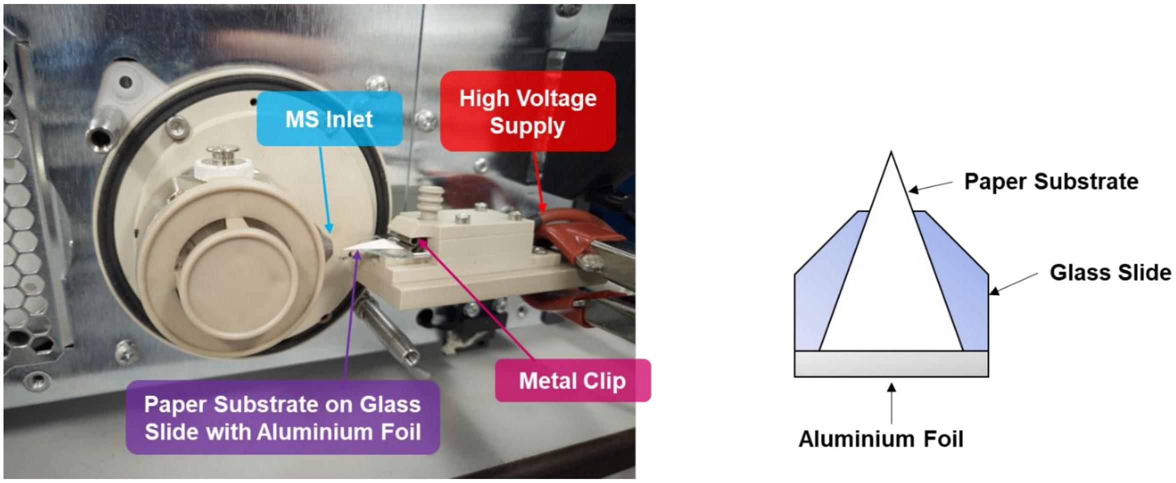


**Figure S1**: Photo on the left shows the in-house constructed paper spray source set up in front of the Waters QDa, with high voltage supply, metal clip and paper substrate on the glass slide with aluminium foil labelled. Schematic on the right shows how the paper substrate is positioned on the glass slide with the aluminium foil.

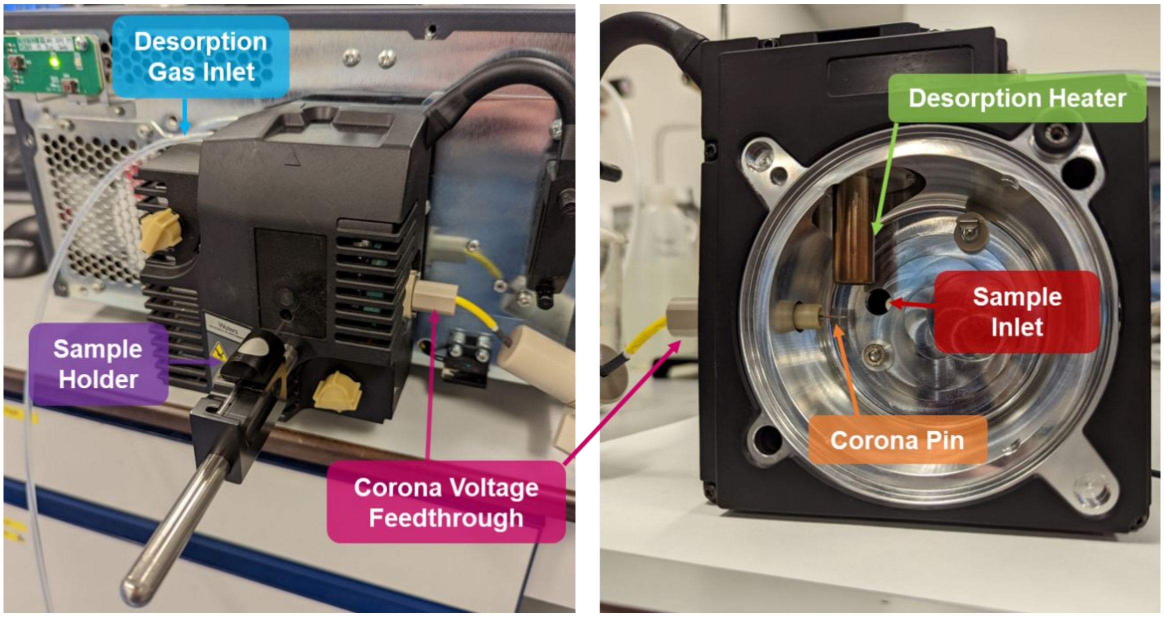


**Figure S2**: Photo of the prototype ASAP source (Waters) coupled to the Waters QDa. Photo on the left hand side shows the desorption gas inlet, sample holder and the corona voltage feedthrough whilst the photo on the right shows the desorption heater, corona pin and where the inserted sample sits. Photo has been adapted from Mathias, S.; Burns, D.; Hambidge, T.; McCullough B.J.; Hopley, C.J.; Douce, D.; Sage, A. Sears, P. Assessment of atmospheric pressure solids analysis probe as a tool for the rapid determination of drug purity. *Drug Test. Anal.* **2023**, DOI: 10.1002/dta.3568.

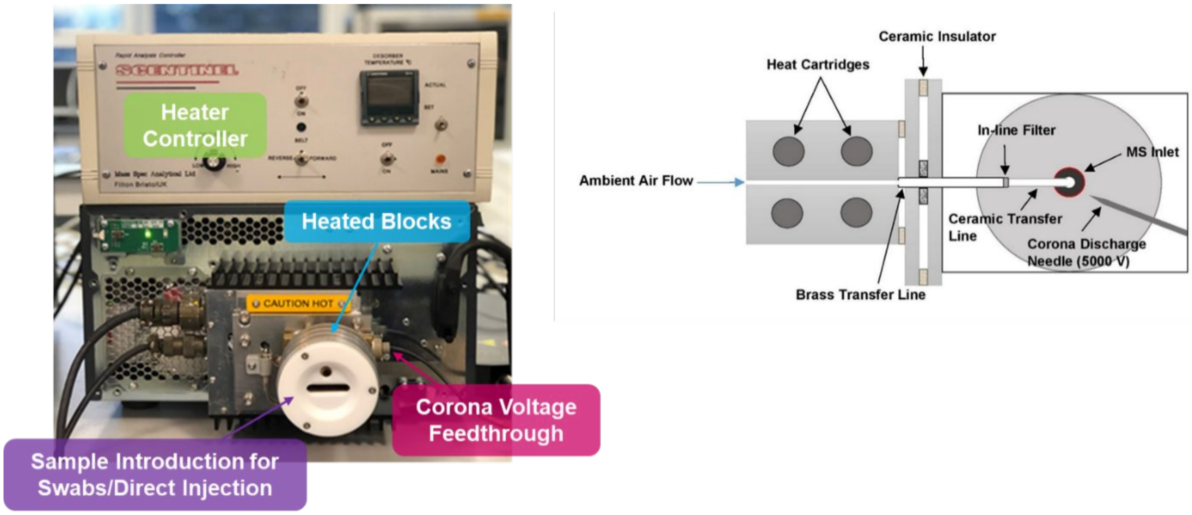


**Figure S3**: Photo on the left shows the prototype TDCD source (Mass Spec Analytical) coupled to the Waters QDa with the heater controller, heated blocks, sample introduction port and corona voltage feedthrough. The labelled diagram to the right (provided by T. Jackson) depicts a cross section of the source.

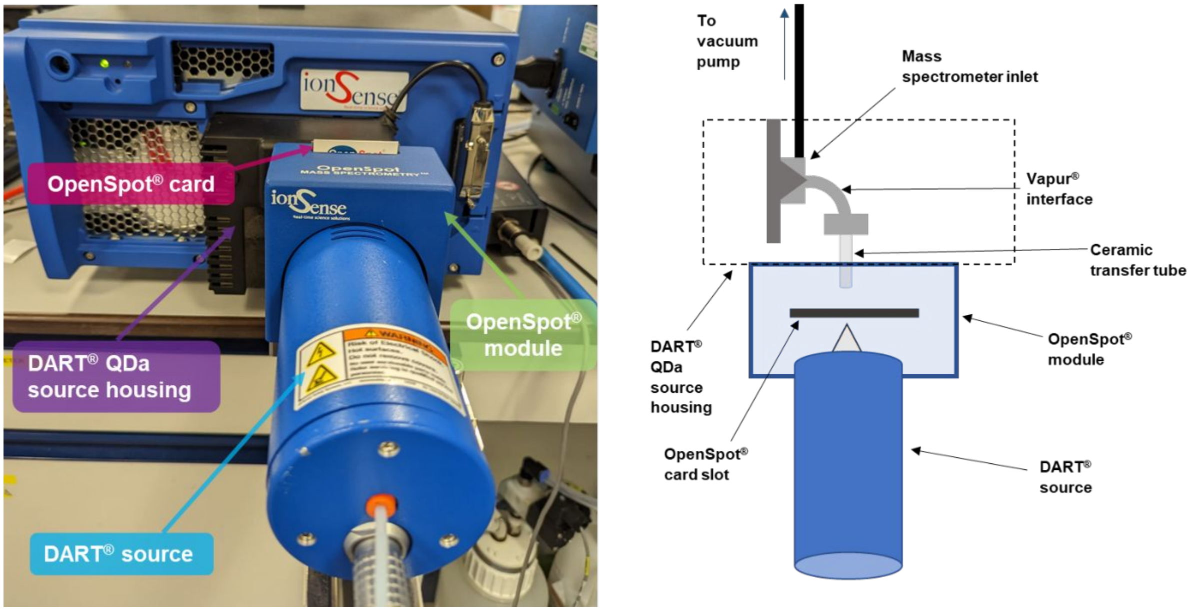


**Figure S4**: Photo on the left shows the DART® source coupled to the Waters QDa accompanied by a labelled diagram on the right. Adapted from Mathias, S.; Sears, P. Direct analysis in real-time mass spectrometry: Observations of helium, nitrogen and argon as ionisation gas for the detection of small molecules using a single quadrupole instrument, *Rapid Commun. Mass Spectrom.* **2023**, 37 (12), e9521. DOI: 10.1002/rcm.9521

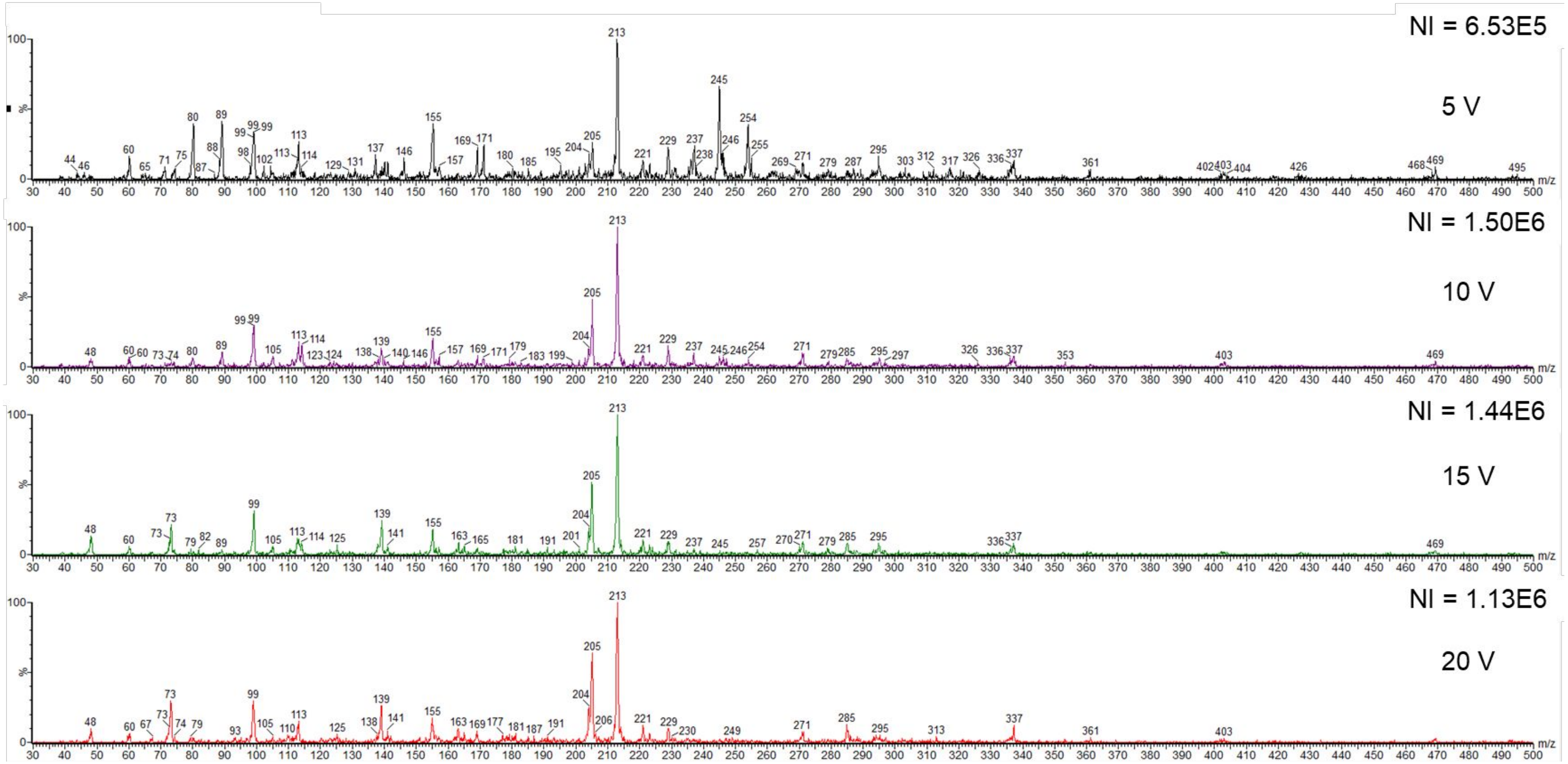


**Figure S5**: Mass spectra of HMTD produced using ESI on the Waters QDa at cone voltages of 5, 10, 15 and 20 V in positive ion mode.

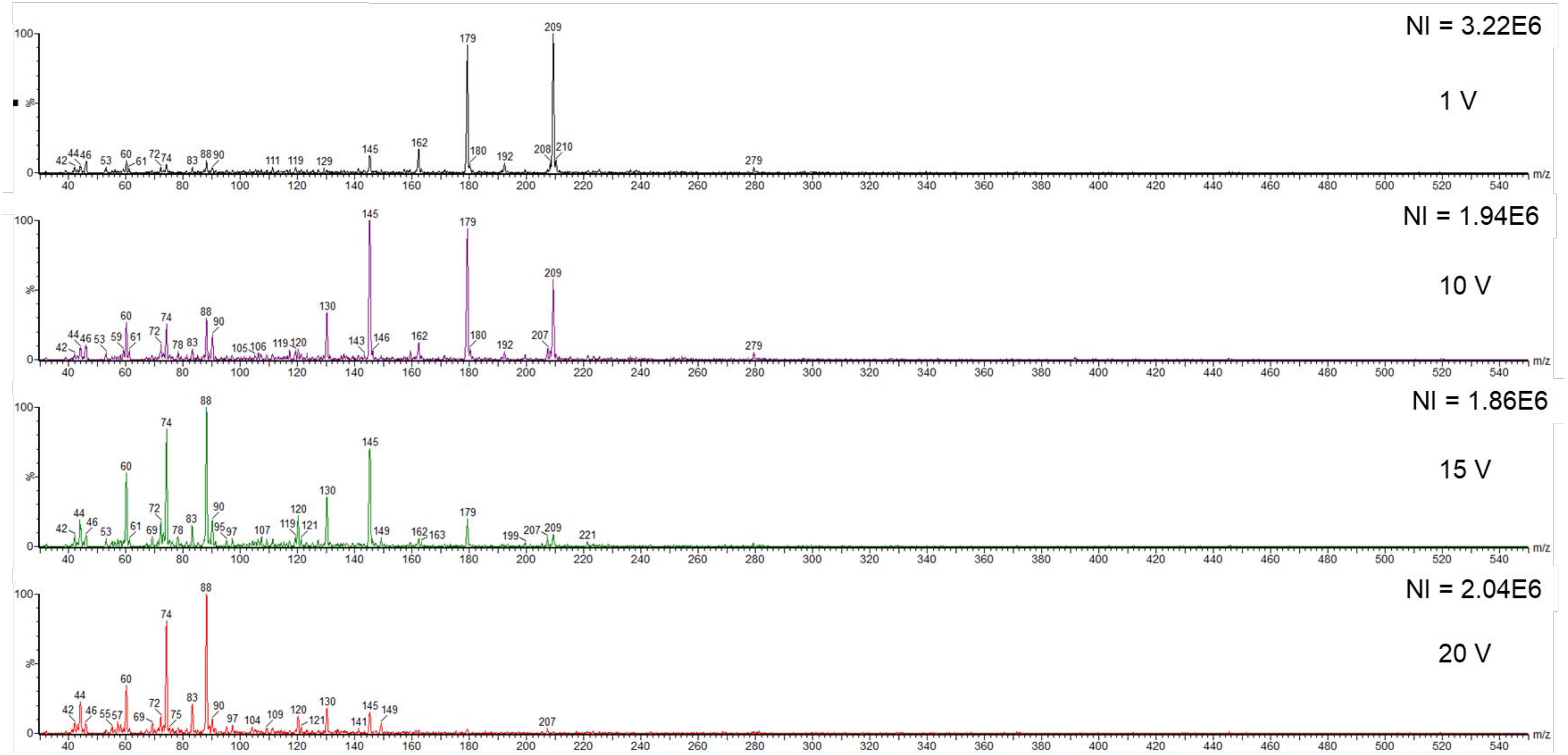


**Figure S6**: Mass spectra of HMTD produced using ASAP (air) on the Waters RADIAN at cone voltages 1, 10, 15 and 20 V in positive ion mode.

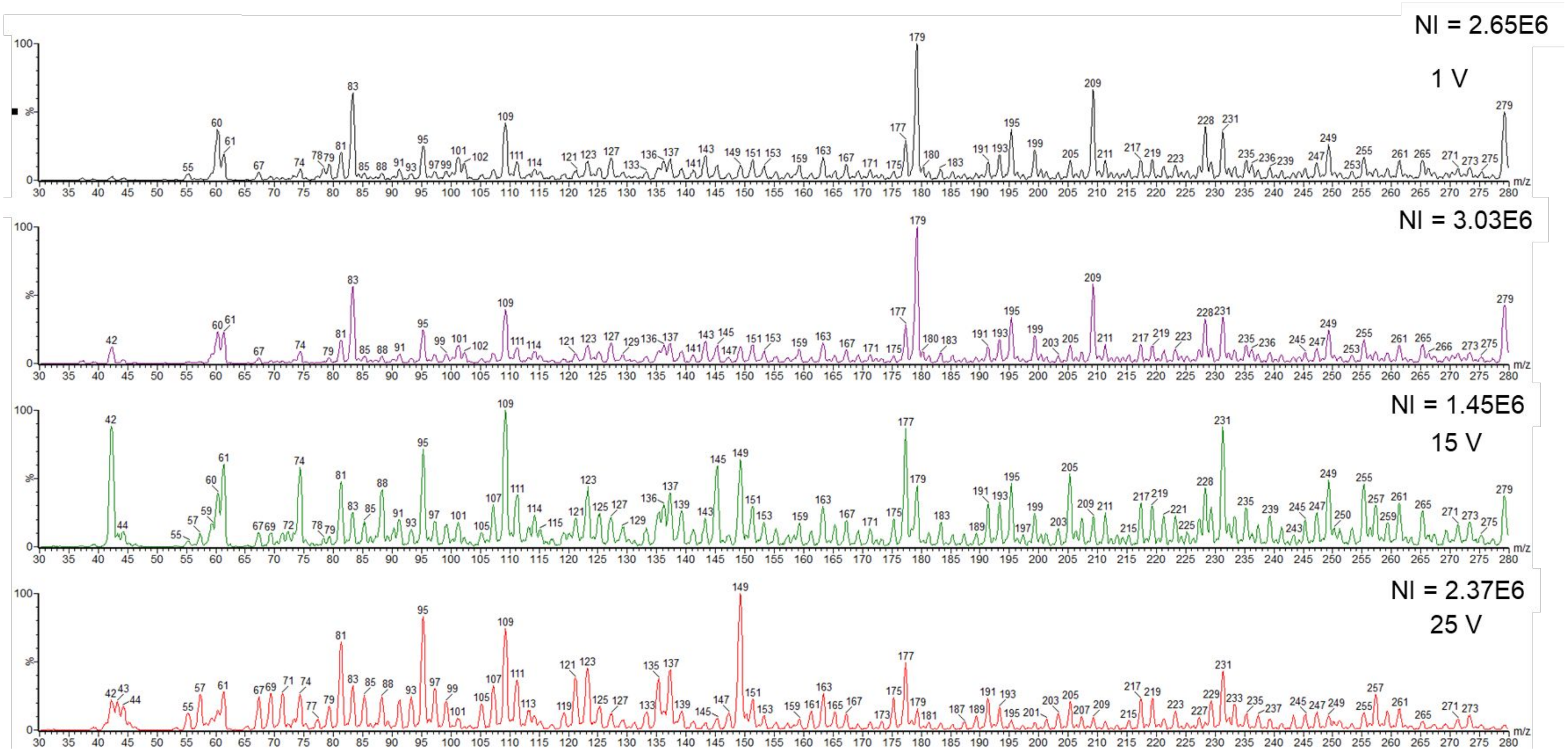


**Figure S7**: Mass spectra of HMTD produced on the TDCD using the Waters QDa at cone voltages at 1, 5, 15 and 25 V in positive ion mode.

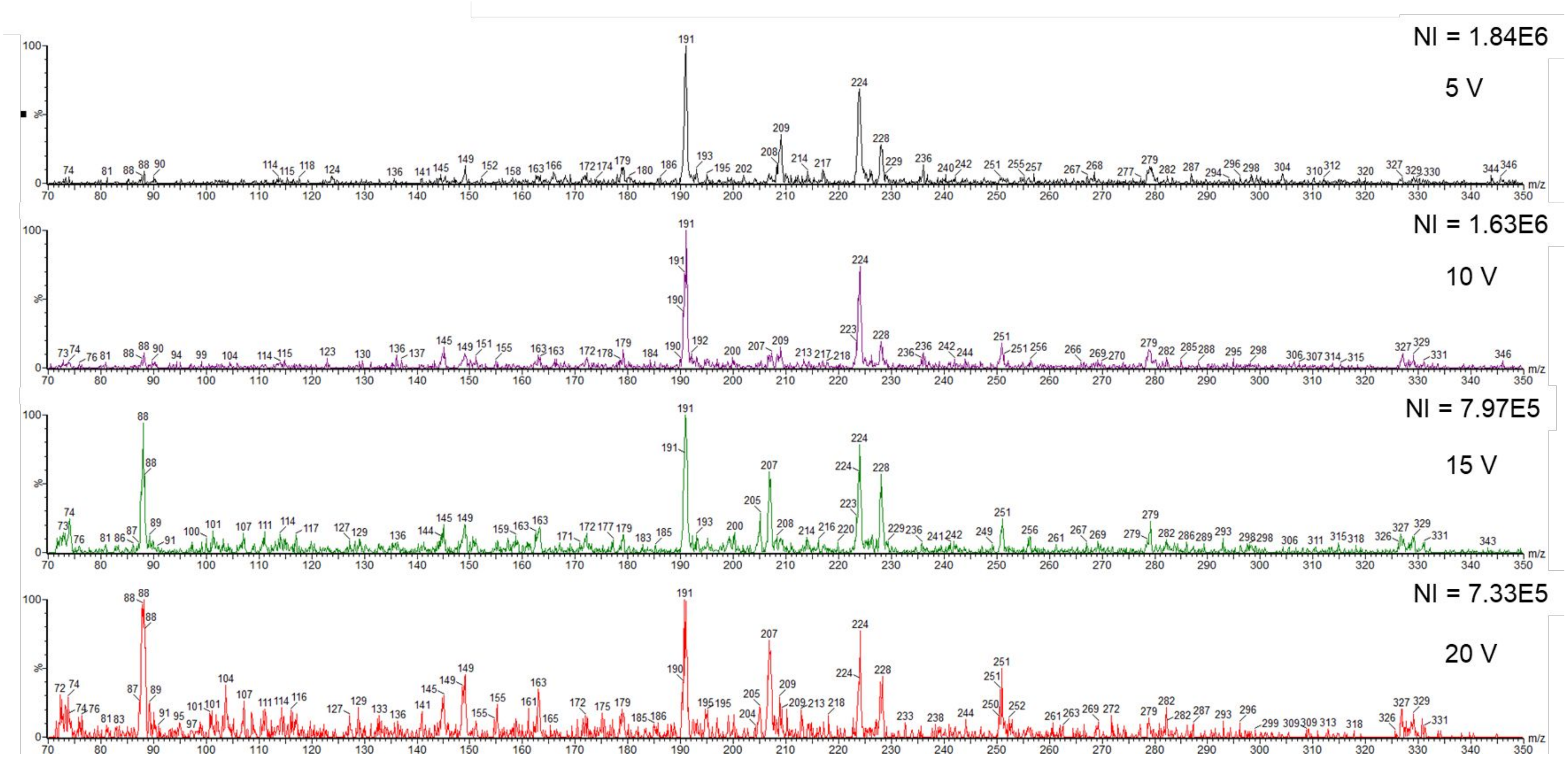


**Figure S8**: Mass spectra of HMTD produced using DART on the Waters QDa at cone voltages 5, 10, 15 and 20 V in positive ion mode.

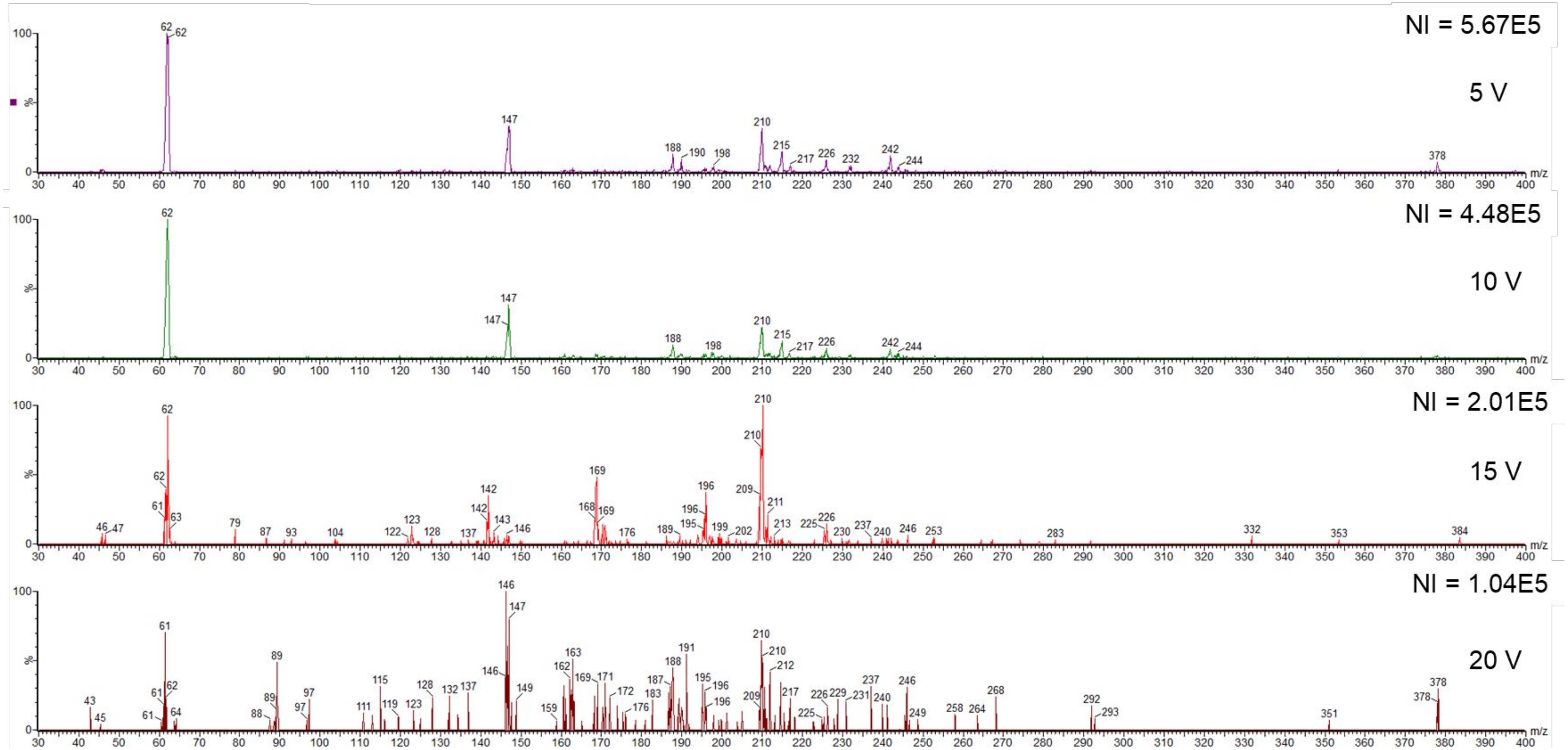


**Figure S9**: Mass spectra of PETN produced using ESI on the Waters QDa at cone voltages 5, 10, 15 and 20 V in negative ion mode.

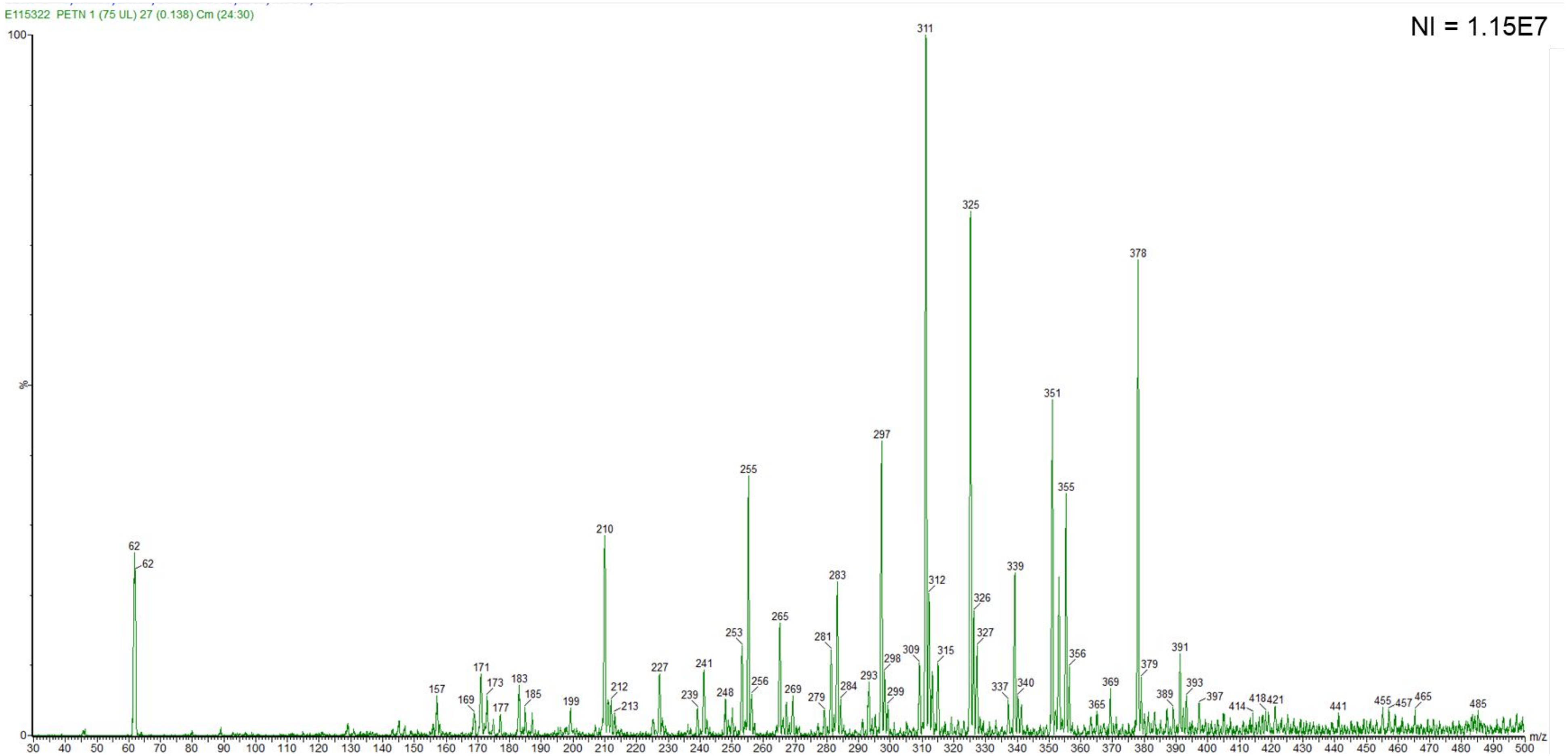


**Figure S10**: Mass spectrum of PETN using paper spray on the Waters QDa at a cone voltage of 5 V in negative ion mode.

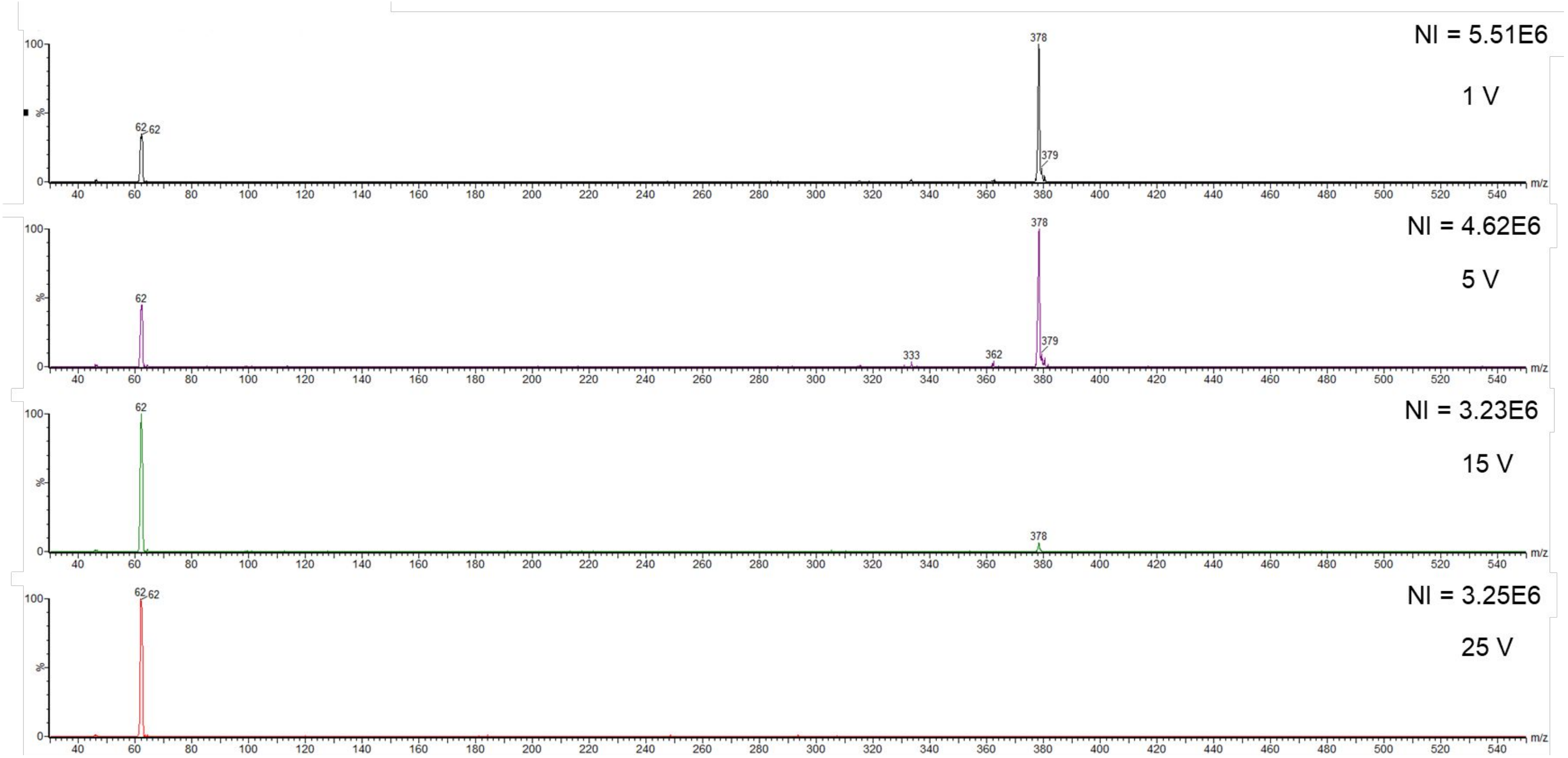


**Figure S11**: Mass spectra of PETN produced using ASAP (nitrogen) on the Waters QDa at cone voltages of 1, 5, 15 and 25 V in negative ion mode.

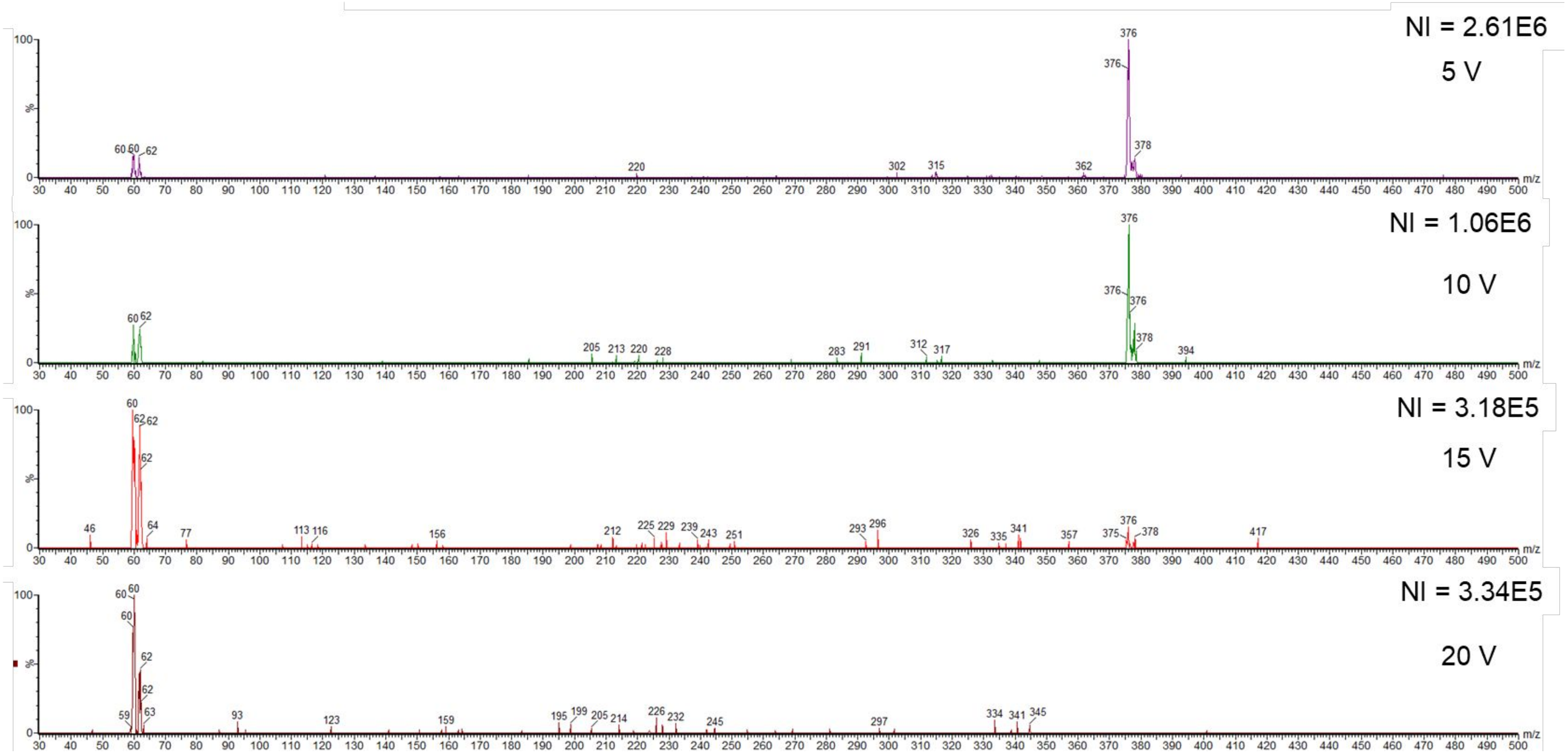


**Figure S12**: Mass spectra of PETN produced using ASAP (air) on the Waters RADIAN at cone voltages of 5, 10, 15 and 20 V in negative ion mode.

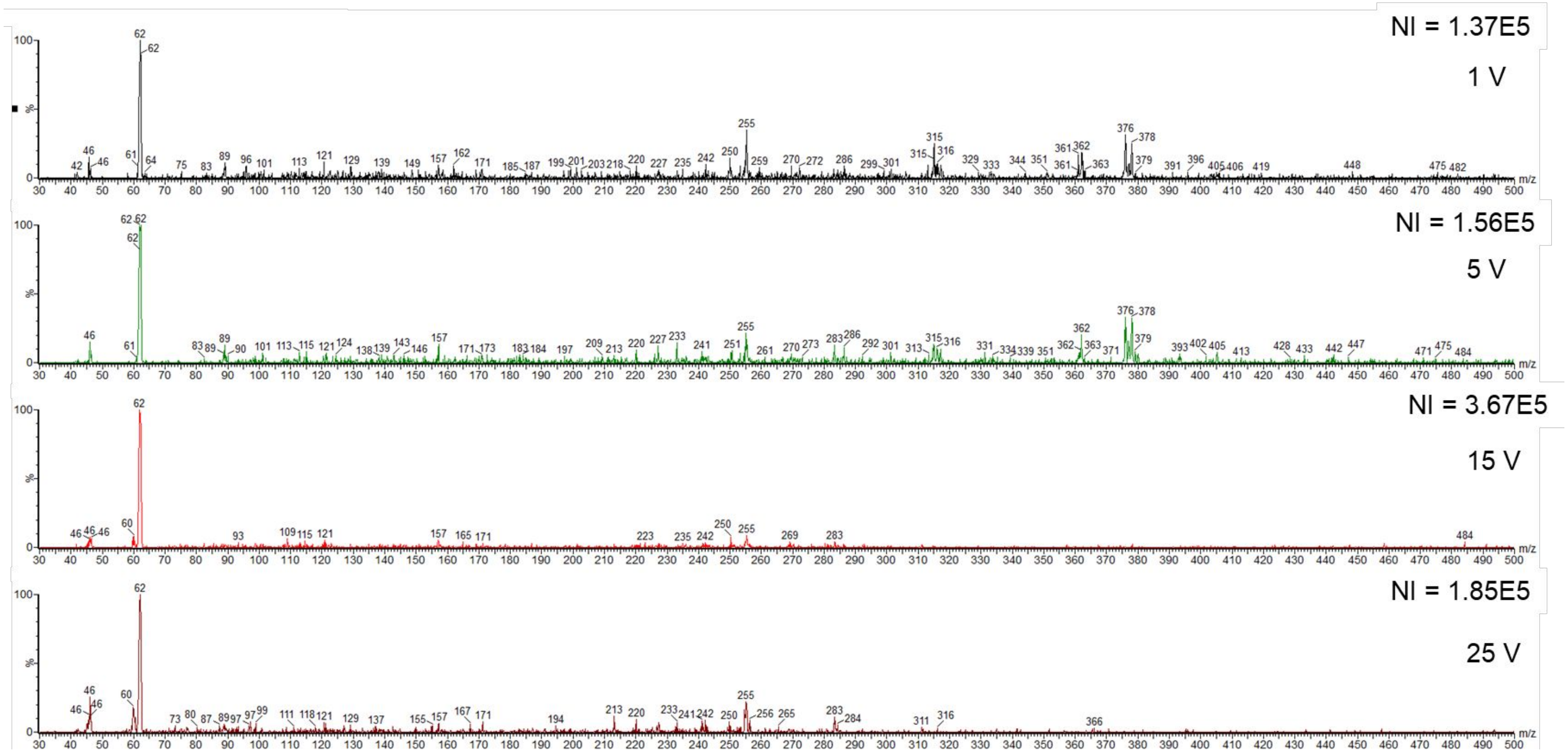


**Figure S13**: Mass spectra of PETN produced using TDCD on the Waters QDa at cone voltages of 1, 5, 15 and 25 V in negative ion mode.

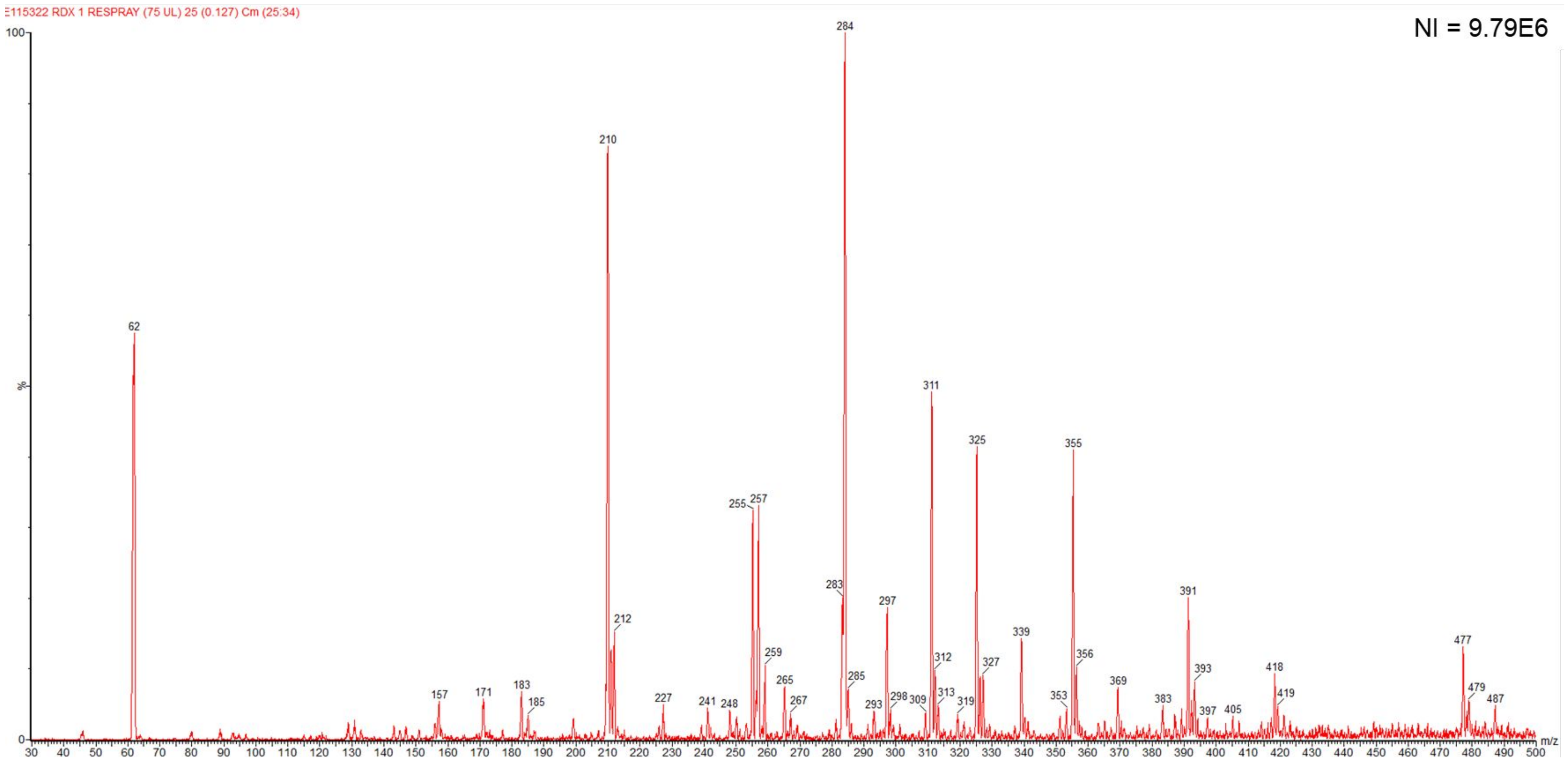


**Figure S14**: Mass spectrum of RDX produced using paper spray on the Waters QDa at a cone voltage of 5 V in negative ion mode.

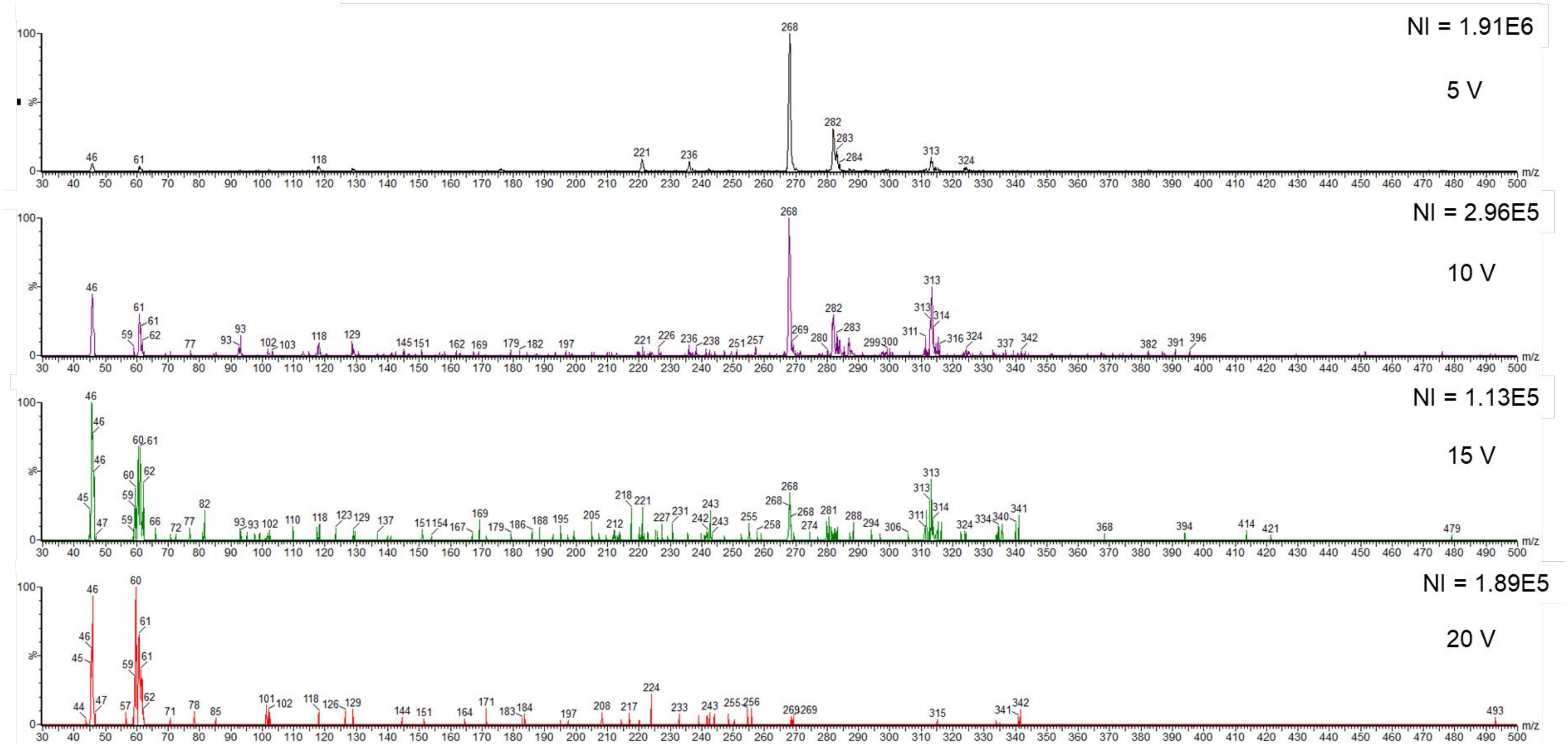


**Figure S15**: Mass spectra of RDX produced using ASAP (air) on the Waters RADIAN at cone voltages of 5, 10, 15 and 20 V in negative ion mode.

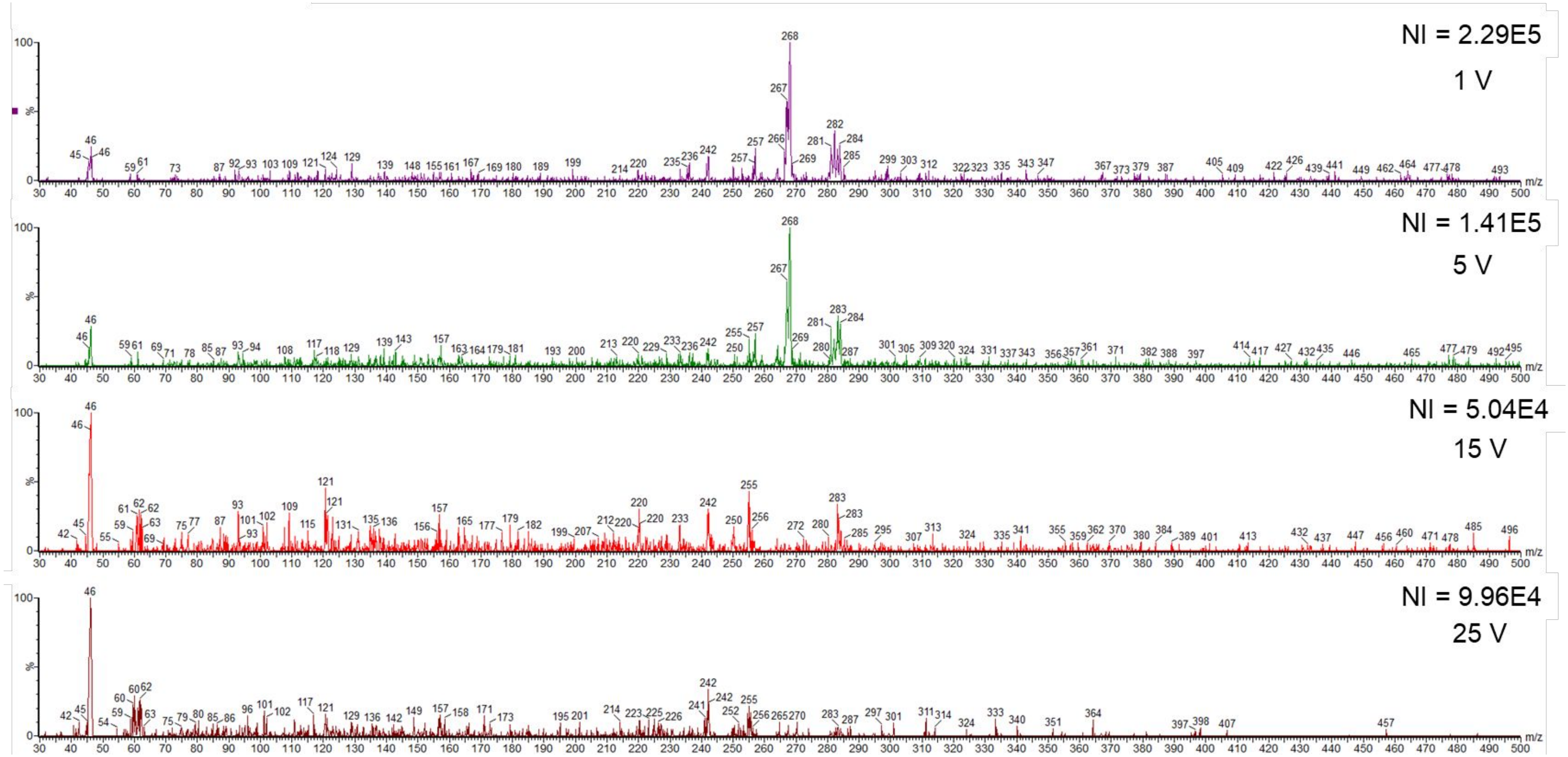


**Figure S16**: Mass spectra of RDX produced using TDCD on the Waters QDa at cone voltages of 1, 5, 15 and 25 V in negative ion mode.

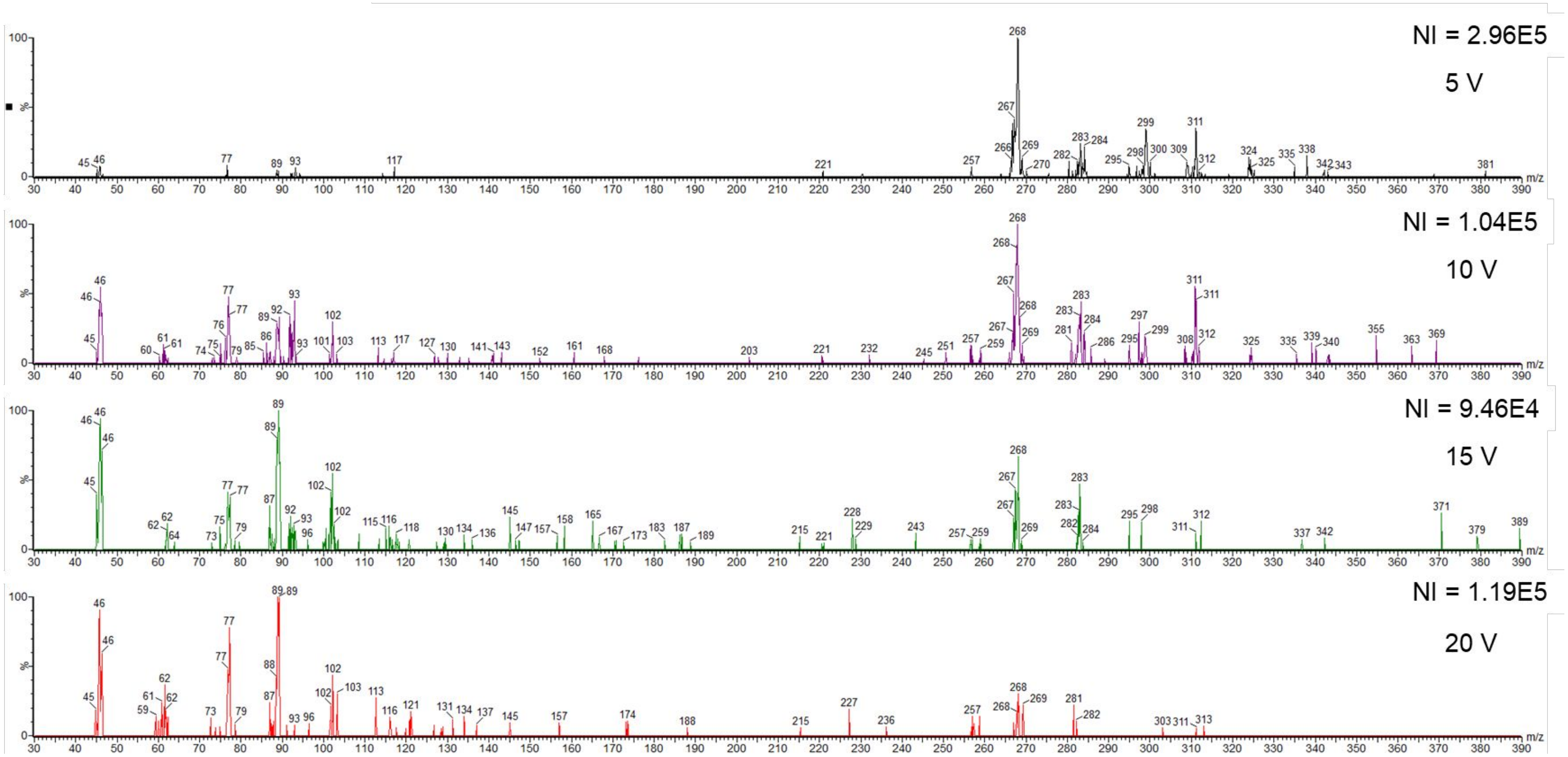


**Figure S17**: Mass spectra of RDX produced using DART on the Waters QDa at cone voltages of 5, 10, 15 and 20 V in negative ion mode.

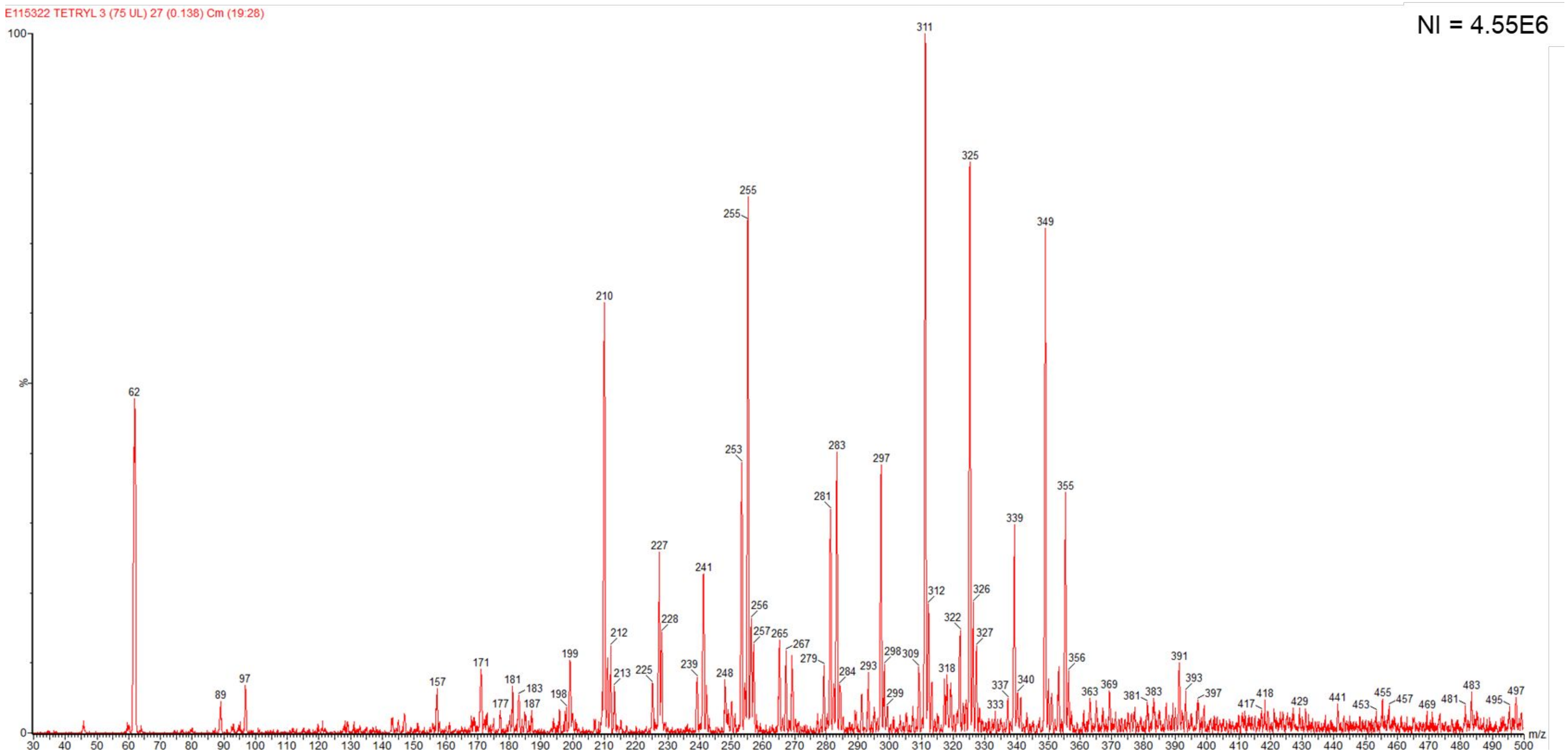


**Figure S18**: Mass spectrum of tetryl produced using paper spray on the Waters QDa at a cone voltage of 5 V in negative ion mode.

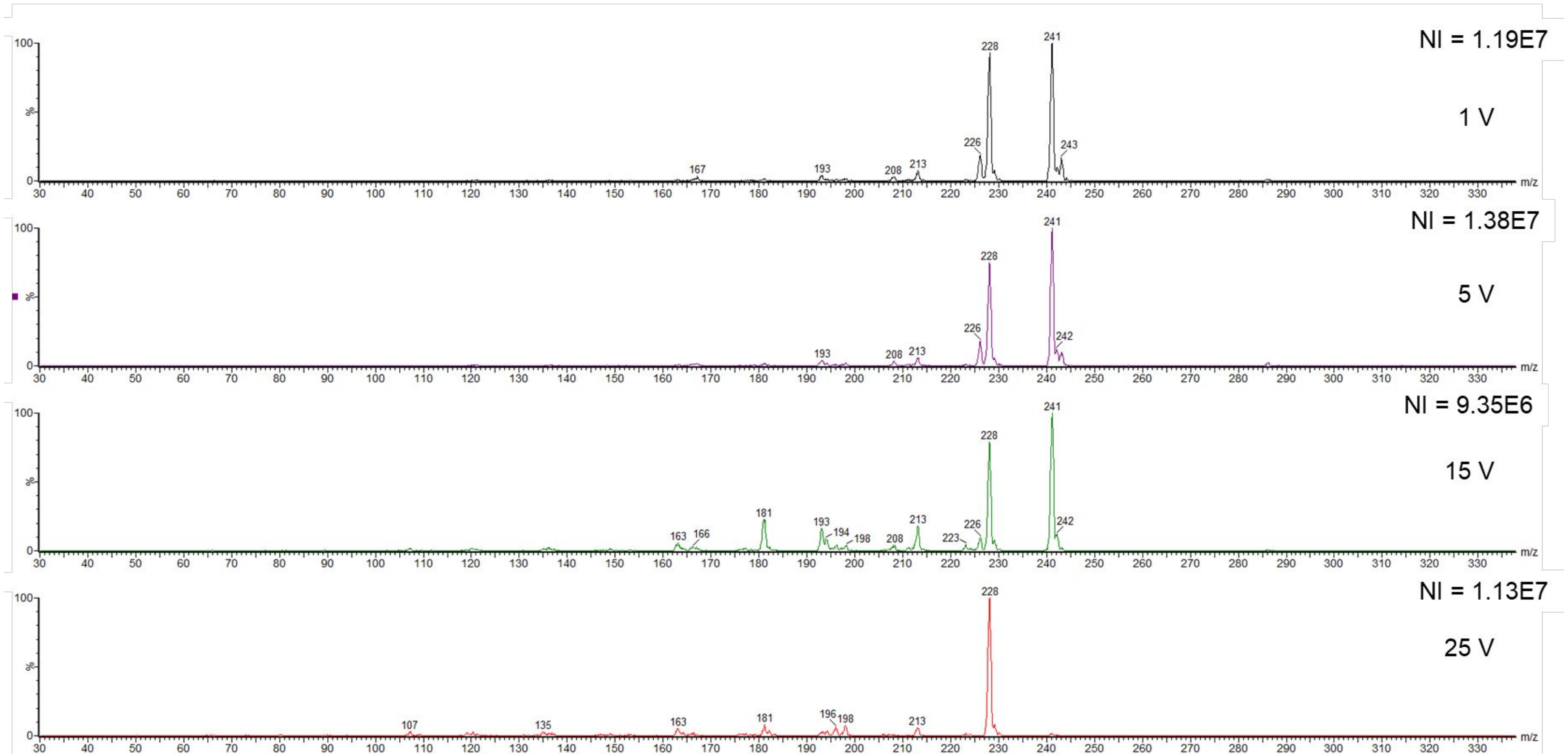


**Figure S19**: Mass spectra of tetryl produced with ASAP (nitrogen) on the Waters QDa at cone voltages of 1, 5, 15 and 25 V in negative ion mode.

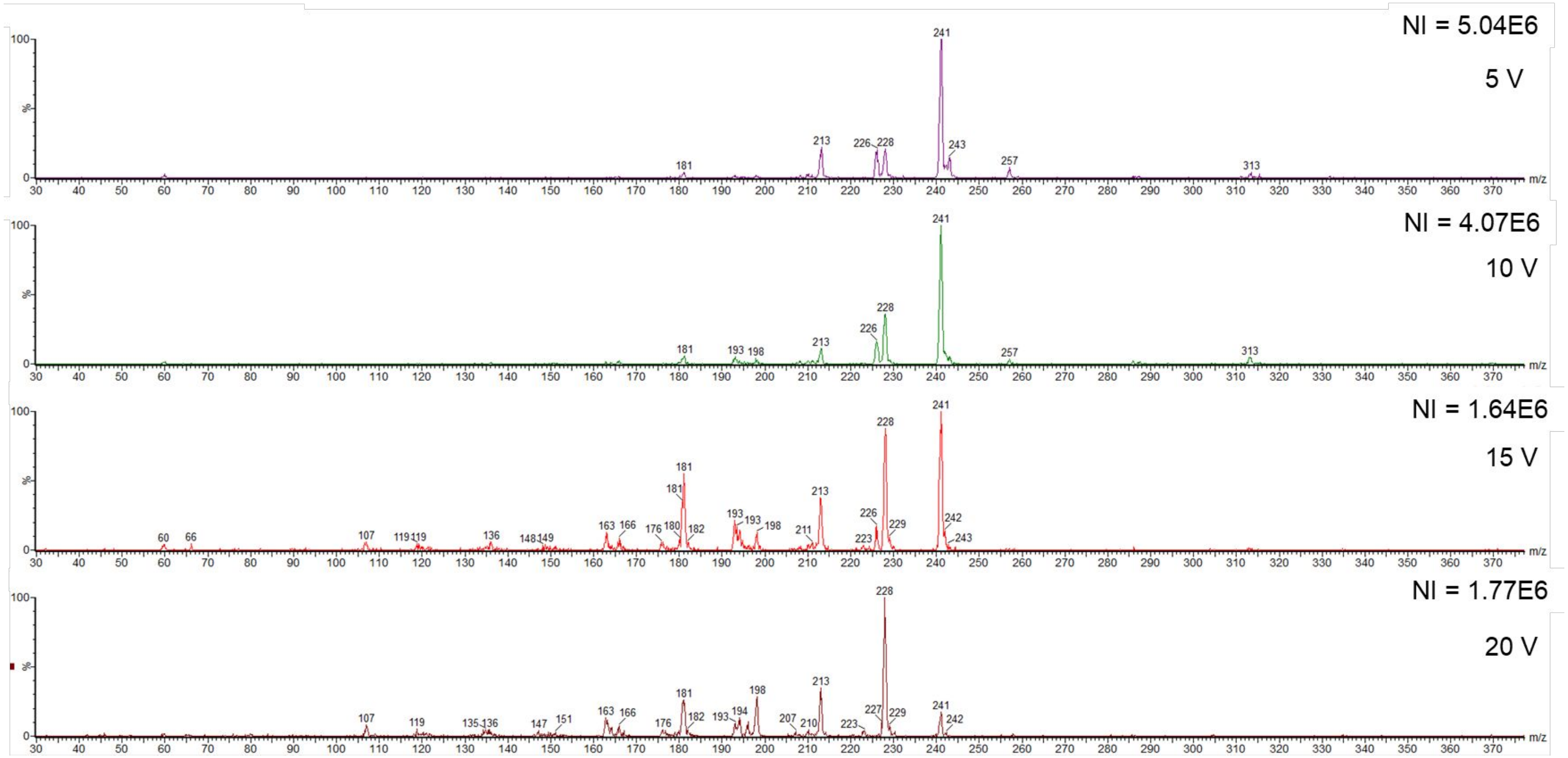


**Figure S20**: Mass spectra of tetryl produced using ASAP (air) on the Waters RADIAN at cone voltages of 5, 10, 15 and 20 V in negative ion mode.

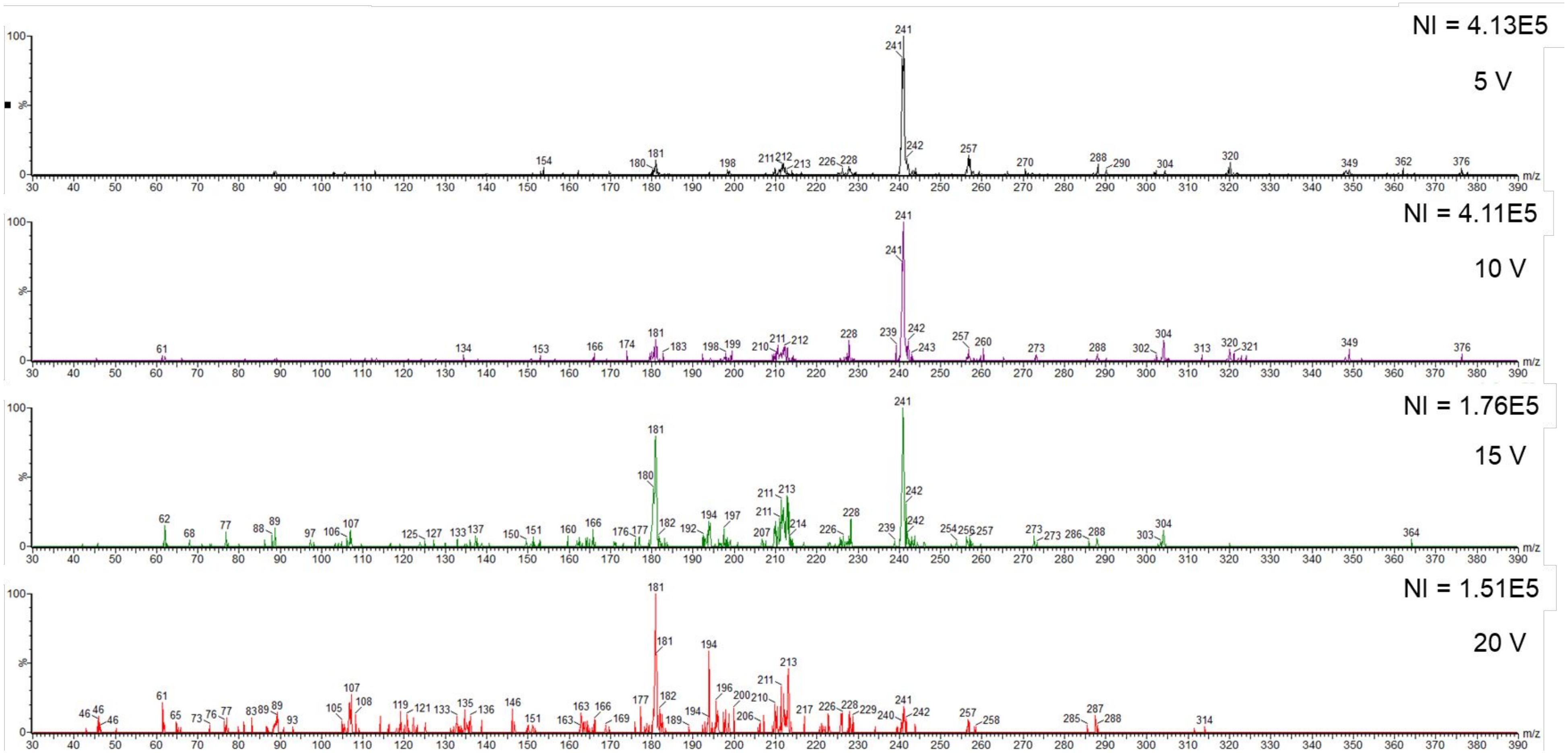


**Figure S21**: Mass spectra of tetryl produced using DART on the Waters QDa at cone voltages of 5, 10, 15 and 20 V in negative ion mode.

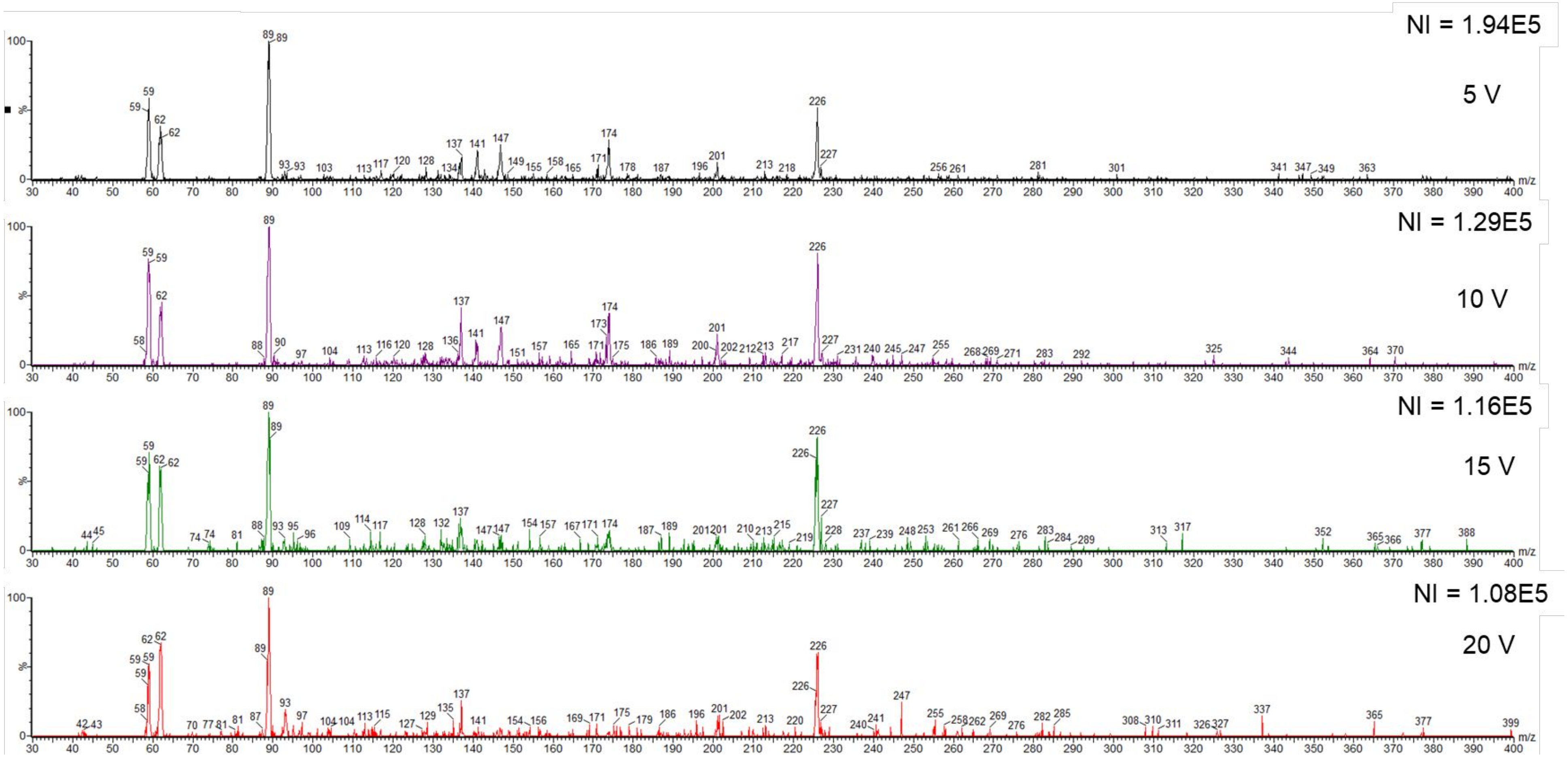


**Figure S22**: Mass spectra of TNT produced using ESI on the Waters QDa at cone voltages of 5, 10, 15 and 20 V in negative ion mode.

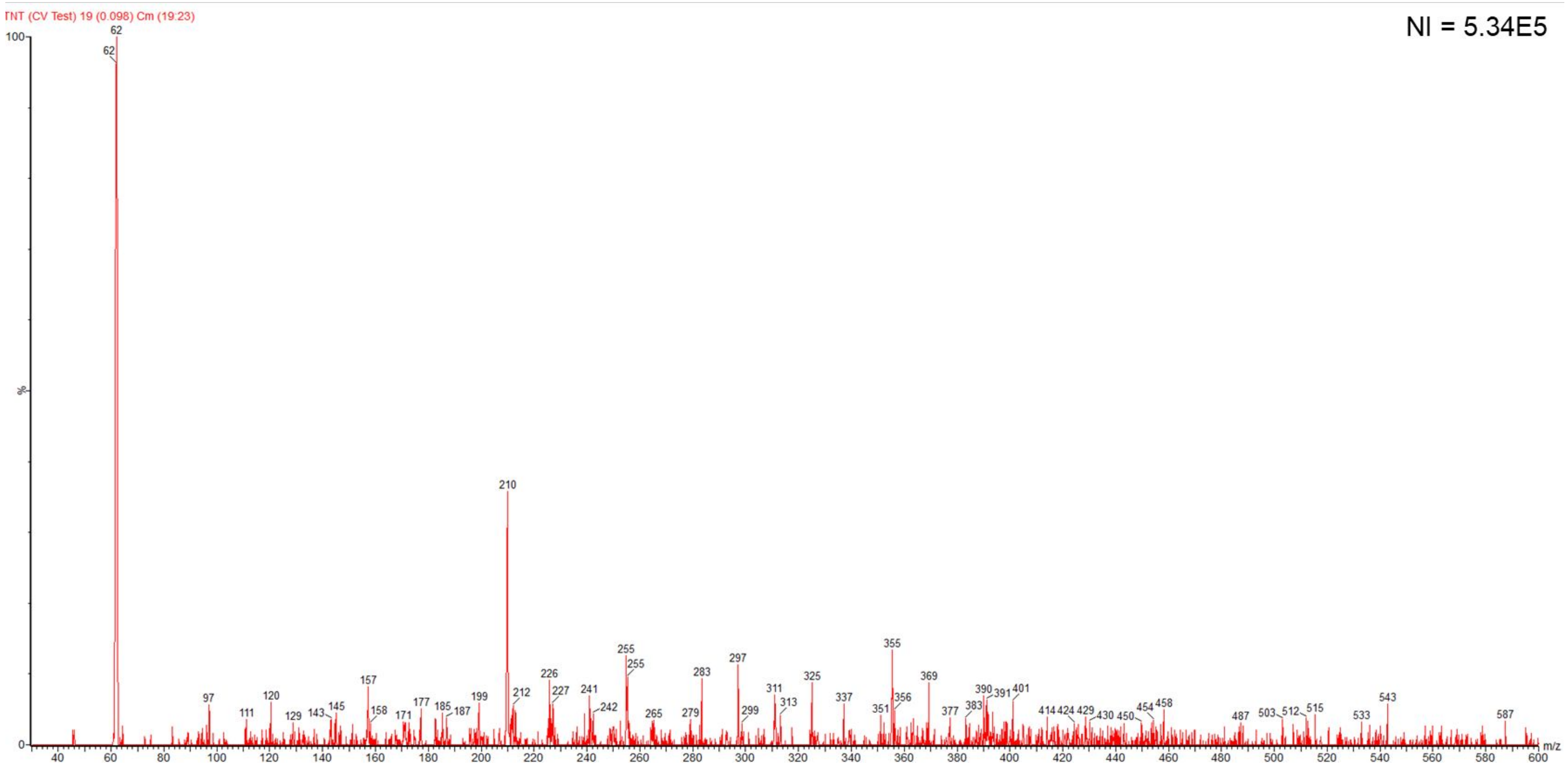


**Figure S23**: Mass spectrum of TNT produced using paper spray on the Waters QDa at a cone voltage of 10 V in negative ion mode.

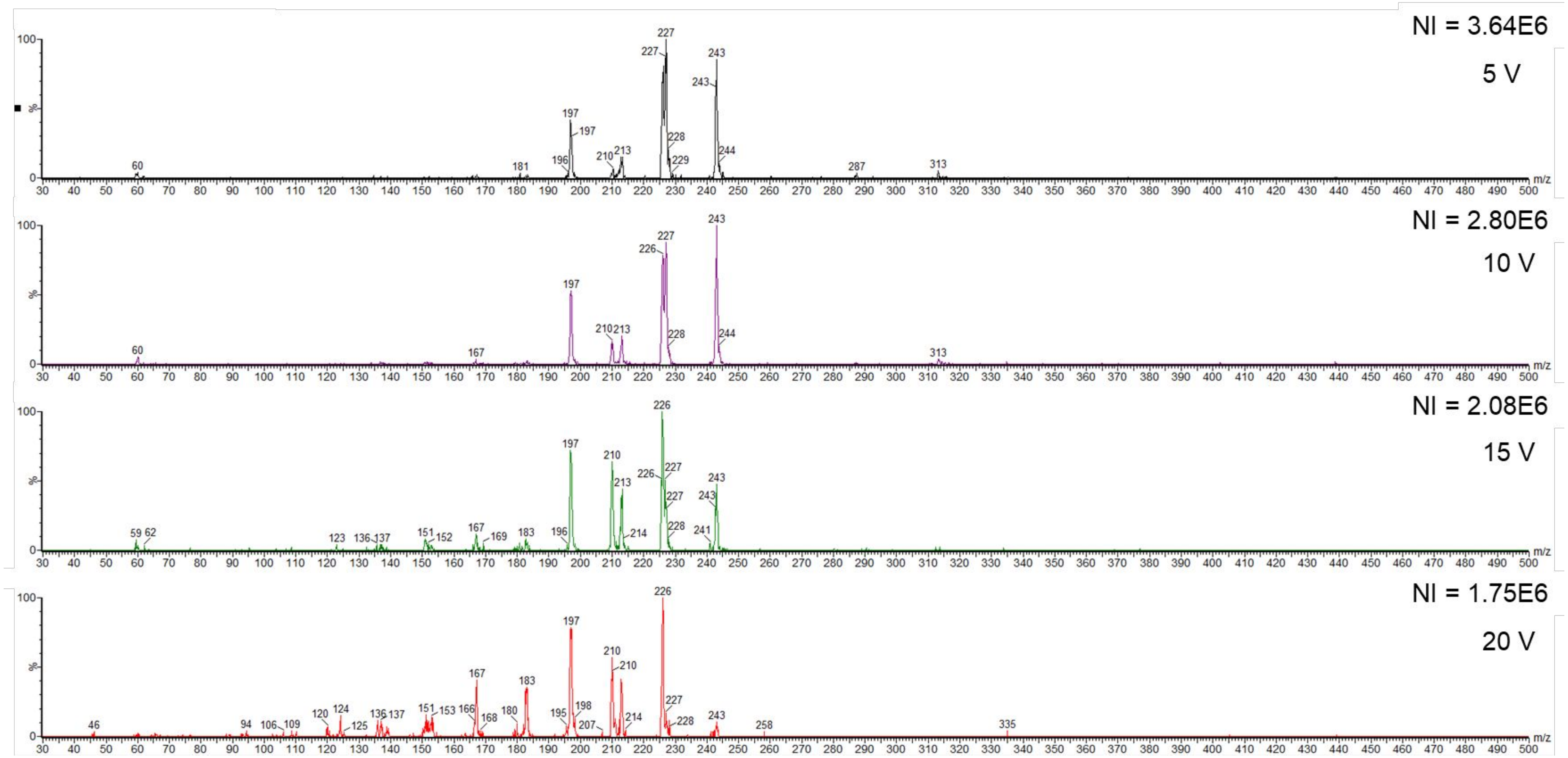


**Figure S24**: Mass spectra of TNT produced using ASAP (air) on the Waters RADIAN at cone voltages of 5, 10, 15 and 25 V in negative ion mode.

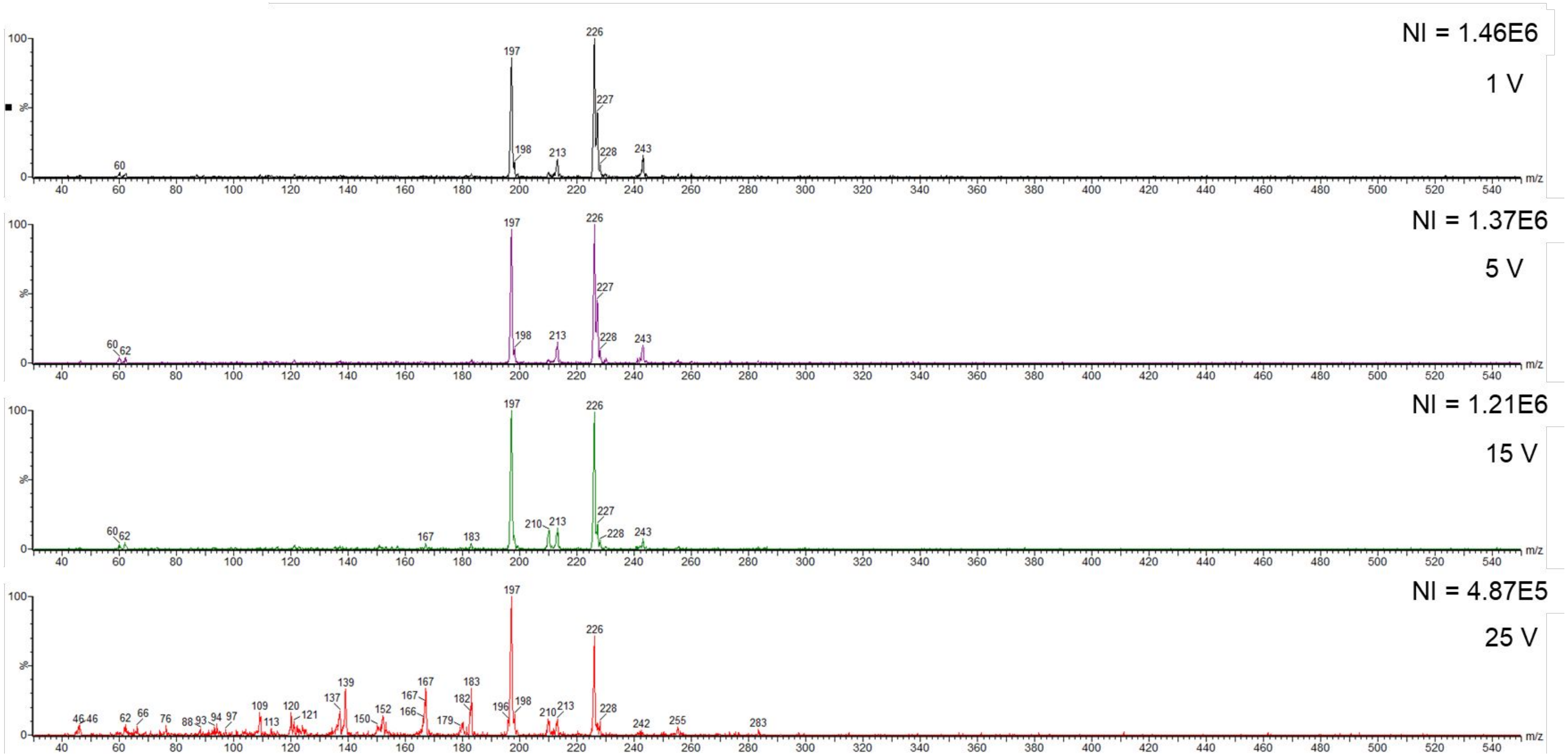


**Figure S25**: Mass spectra of TNT produced using TDCD on the Waters QDa at cone voltages of 1, 5, 15 and 25 V in negative ion mode.
